# Supplementary material for: RETurn to work After stroKE (RETAKE) Trial: protocol for a mixed-methods process evaluation using normalisation process theory
Source: BMJ Open. 2022 Mar 15;12(3):e053111. doi: 10.1136/bmjopen-2021-053111 (PMC8928258; doi:10.1136/bmjopen-2021-053111)
Supplement: Supplementary data [file bmjopen-2021-053111supp001.pdf]

**Appendix I**	**ESSVR Description (TIDieR)**
**Brief Name** (Provide the name or a phrase that describes the intervention.)	1a) Early Stroke Specific Vocational Rehabilitation (ESSVR)<br>1b) The Return to Work after Stroke (RETAKE) trial
**WHY** Describe any rationale, theory, or goal of the elements essential to the intervention.	**Rationale**<br><br>Stroke is common (>100,000 strokes per annum in the UK) [1]. In spite of reperfusion therapy and secondary prevention, outcomes remain poor - almost two-thirds of survivors leave hospital with a disability, and a third experience depression and/or cognitive impairment. Stroke survivors of working age are 2-3 times more likely to be unemployed [1].<br><br>Increasingly, there is an expectation that existing health and social care pathways for stroke survivors provide support for stroke patients intending to return to work [2-9]. Despite improvements in the organisation of stroke rehabilitation services following discharge, many stroke survivors fail to access this support because a) their work rehabilitation needs are not identified early after stroke b) many have hidden disabilities such as visual or cognitive impairments and fatigue, which are missed in the acute phase [10] and c) the criteria for referral to community rehabilitation are impairment based rather than needs led, meaning that a person with unmet needs for work participation alone (rather than a need for support from more than one healthcare professional e.g. Occupational Therapy and Speech and Language Therapy) may be unable to access support. d) Not all community stroke services provide rehabilitation that addresses work needs [11]. Where they do this may be time limited or fail to engage with employers in the workplace, as supporting a return to work is not always seen as the job of health [9]. Furthermore, stroke survivors themselves may not appreciate the true impact of the stroke on their workability until they attempt to return to work [12].<br><br>Failure to provide this support, may lead to job loss, affecting physical, emotional, and financial wellbeing and quality of life [13,14]. Return to work is a recognised outcome of health interventions [15]. Supporting people who develop health conditions to return to work is recommended in stroke policy and clinical guidelines [3,4,5,7].<br><br>The UK government has committed to reduce the employment gap (54% Vs 82%) between disabled and non-disabled people. Its goal is to see one million more disabled people in work by 2027 [16].<br><br>The Equality Act requires employers to make reasonable adjustments, to accommodate the person in the workplace [17]. These adjustments may involve more breaks, reductions in working hours, reduced responsibilities, increased supervision, flexible working patterns and working from home and help from other people or agencies, including rehabilitation.<br><br>**The 'theory of change underpinning ESSVR'**<br><br>Health based preparation and support for returning to work after stroke has typically been deficient in meeting stroke survivors work needs. ESSVR was designed to bridge the gap between existing stroke rehabilitation services, the employment and the voluntary sector in supporting stroke survivors in a return to work [10] Tested in a single centre feasibility trial we found evidence to suggest that that the intervention may have potential to support job retention at 12 months post stroke [18].<br><br>The implicit theory of change on which ESSVR can be expressed as follows:

	Stroke brings about physical and psychological impairments that are likely impact on the capacity to return to and remain in work<br><br>The ability to identify work needs early in the stroke pathway is missing from stroke services and vocational rehabilitation knowledge and skills gap is present in stroke rehabilitation services. Implementing mechanisms for identifying stroke survivors who are employed at stroke onset; educating the stroke care team about 'return to work' and teaching OTs with stroke specific knowledge basic skills in vocational rehabilitation, disability discrimination, how to evaluate jobs and assess work capability and match stroke survivor's abilities to job demands;<br>how to engage with employers, and other employment sector stakeholders, to go into the workplace and how to negotiate reasonable adjustment and phased return to work will enable stroke services to support stroke survivors in a return to work.<br><br>The logic model (Figure 1) has the following underlying assumptions;<br><br>• *If* we implement an early 'VR pathway' for stroke *then, w*ork is seen as a health outcome by stroke rehabilitation teams, conflicting advice prevented, increased confidence, knowledge and skills in VR, patient aware of available support & how to access; Early barriers to RTW identified e.g. environmental (job type), personal. Recognising work as an outcome of health interventions thus promoting a shared philosophy of rehabilitation to support return to work **[Mechanism: Early Intervention, Collective Understanding]**<br><br>• *If* we identify people who are employed at the time of stroke and refer to an Occupational Therapist trained in VR (VR OT) for information/advice/ support re return to work (RTW), *then* this will increase opportunities for RTW & prevent job loss; prevent people from falling into service gaps, and ensure work needs are met. [**Mechanism: Early Identification]**<br><br>• *If we* teach OTs basic skills in vocational rehabilitation (how to evaluate jobs and assess work capability, match the injury related disabilities to job demands; how to engage with employers, and other employment sector stakeholders, go into the workplace and how to negotiate reasonable adjustment and a phased return to work) *then* they will have the confidence, knowledge and skills to support stroke survivors in a return to work **[Mechanism: VR Upskilling; Clinicians confident and empowered; Assessment]**<br><br>• *If the OT* provides early (within 8 weeks of stroke) assessment, education and advice on the impact of stroke & RTW, *then* the impact of the stroke on the job role will be identified to inform a vocational rehabilitation plan. Persons requiring psychological support for mental health issues are identified and referred for support, resulting in improved physical and mental health and financial wellbeing. **[Mechanisms: Assessment; Education Early intervention]**<br><br>• *If the OT* delivers individually tailored vocational rehabilitation, engaging with the employer to negotiate workplace accommodations, a phased return to work, educating employers and monitors ongoing work ability, *then,* the person will be able to cope with work, resulting in reduced sickness absence and sustainable employment. **[Mechanisms: Individual Tailoring; Accommodating stroke at work, Colocation, Employer Engagement, communication]**<br><br>ESSVR is a biopsychosocial intervention informed by the International Classification of Function (ICF) [19] and the 'Work Disability Arena' or Sherbrooke model [20]. It takes into consideration the overall context of an individual. It identifies the level of functioning at the body, person and societal level, as well as understanding the personal and environmental contextual factors that may impede or enhance work participation.

	It aims to prevent job loss by drawing on employment law and the Equality Act (2010) (17) to prevent disability discrimination and ensure "reasonable adjustments" are negotiated with employers to reduce the impact of stroke disability by accommodating (modifying) the stroke survivor's job to enable a return to work. ESSVR also ensures patients are provided with appropriate individualised work-related physical and cognitive rehabilitation and self-management education to increase their ability to work.
**WHAT**<br><br>**Materials**: Describe any physical or informational materials used in the intervention, including those provided to participants or used in intervention delivery or in training of intervention providers. Provide information on where the materials can be accessed (e.g. online appendix, URL).<br><br>**Procedures**: Describe each of the procedures, activities, and/or processes used in the intervention, including any enabling or support activities.	**Materials:**<br><br>**Training:** Occupational therapists are provided with an 'ESSVR Intervention manual' detailing the intervention content, its rationale and objectives, processes to be followed and forms for use in documenting ESSVR delivery in the trial. The manual included examples of return to work plans, sample graded RTW planning, session and work review letters, sample letters to GP, discharge letters, letter to employer, sample report for occupational health and a list of other useful resources (below). The manual was sent to therapist two weeks before the training and used during the training to navigate them through the ESSVR intervention process and familiarise them with its contents and resources.<br><br>**Resources included:**<br><br>For Occupational Therapists<br><br>• Employment and Support Allowance (ESA) Supporting letter and Guide to completing ESA (2012), See 50 9 esa50guide2012 (nawra.org.uk)<br>• Allied Health Professions Fitness For Work Report (RCOT), Accessible via https://www.rcot.co.uk/practice-resources/standards-and-ethics/ahp-health-and-work-report<br>• AHP Health and Work Report: Guidance for AHP practitioners on the use and completion of the Report (Allied health Professions Federation). See; Guidance-on-completion-of-AHP-Health-and-Work-Report.pdf (ahpf.org.uk)<br>• Graded RTW planning leaflet (RETAKE Trial specific)<br>• Tailored Adjustments Plan (Business Disability Forum, 2020) Accessible via Tailored Adjustments Plans - Business Disability Forum<br>• Work Ability Support Scale (WSS) (Fadyl J, McPherson KM, Schulter P, Turner-Stokes L., 2014) [21] Accessible via https://www.kcl.ac.uk/cicelysaunders/resources/tools/wss<br>• WSS Detailed work questionnaire, Accessible via https://www.kcl.ac.uk/cicelysaunders/resources/tools/wss<br>• WSS Brief work questionnaire and jobe matching, Accessible via https://www.kcl.ac.uk/cicelysaunders/resources/tools/wss<br>• THE CITY OF TORONTO S JOB DEMANDS ANALYSIS AND JOB MATCH SYSTEM (Lucas, 2017), accessible via; https://silo.tips/download/the-city-of-toronto-s-job-demands-analysis-and-job-match-system<br>• Beginners Guide to Benefits, Accessible via https://www.turn2us.org.uk/Benefit-guides/Beginner-s-Guide-to-Benefits/Checking-benefit-entitlement<br>• Good work for good health The difference occupational therapy makes, (RCOT, 2019) Accessible via ILSM Work report A4 7pp D7.pdf (rcot.co.uk)<br><br>For Employers<br><br>• Employees with Executive Functioning Deficits (Job Accommodation Network 2018) , Accessible via; Brain Injury (askjan.org)<br>• Accommodation and Compliance Series: Employees with Speech-Language Impairment (Job Accommodations Network, 2019) Accessible via JAN-Job-accomadation-suggestions.pdf (dysphonia.org)

	• Job accommodations for people with motor limitations from stroke (Morgantown, WV, Office of Disability Employment Policy, Job Accommodation Network, 2010) Accessible via Job accommodations for people with motor limitations from stroke - University of Missouri Libraries<br>• A complete guide to stroke for Employers (Stroke Association, 2019), See: f41cg_a_complete_guide_to_stroke_for_employers_v3_oct_2019.pdf,<br>• Information Pack -Work After Stroke - Information for Employers, (Different strokes, 2018) Available at: Work After Stroke (differentstrokes.co.uk)<br><br><u>For stroke survivors</u><br><br>• Information Pack Work After Stroke - Information for Family & Friends (Different Strokes, xxx year) Accessible via: Work After Stroke - Information for Family & Friends<br>• A_complete_guide_to_work_and_stroke.pdf See: Your rights at work after stroke | Stroke Association, (Stroke Association, UK)<br>• Driving after a Stoke guide; (Stroke Association, 2021) See f02_driving_v_3.1_web_june_21.pdf (stroke.org.uk)<br>• Stroke in people of working age (Stroke Association, 2014), Accessible via: stroke_in_people_of_working_age.pdf<br>• Tailored Adjustments Plan (Business Disability Forum, 2020) Accessible via Tailored Adjustments Plans - Business Disability Forum<br><br>**Links provided to other Online Resources**<br><br>**Advisory services**<br>• ACAS- Advisory, Conciliation and Arbitration Service- provides support in assisting employment disputes including those related to disability management: http://www.acas.org.uk<br>• Citizens Advice Bureau: http://www.citizensadvice.org.uk/<br>• Disability Law Service: www.dls.org.uk<br>• Disability Rights UK http://disabilityrightsuk.org/<br>• Equality and Human Rights Commission http://www.equalityhumanrights.com/<br><br>• **Occupational Health Advisory Service** – Fit for Work offers free, expert and impartial advice to anyone looking for help with issues around health and work. You can browse our online resources, chat online to a specialist advisor, email a question or call our free advice line on 0800 032 6235 (English) or 0800 032 6233 (Cymraeg). https://fitforwork.org/<br><br>**Details of occupational health providers**<br>• Occupational health support can be very helpful in complex cases Occupational health services are sometimes provided by NHS or local authority services. To find details of providers in your area, contact:<br>• Commercial Occupational Health Provider Association www.cohpa.co.uk<br>• NHS Health at Work www.nhshealthatwork.co.uk/support-for-business.asp<br>• Society of Occupational Medicine www.som.org.uk<br>• Safe Effective Quality Occupational Health Service (list of approved occupational health providers) http://www.seqohs.org<br><br>**Job Centre Plus:**<br>• Disability Employment Advisers are based in Jobcentres, and work with claimants facing complex employment situations because of a disability or health condition. They can act as an advocate with prospective employers if necessary, aiming to identify work solutions that will overcome or minimise any difficulties related to an individual's disability in the work place. https://www.gov.uk/specialist-employability-support<br>• Welfare Benefits and Department for work and Pensions (DWP)<br>• Benefits (including Attendance Allowance, Employment Support Allowance, and Disability Living Allowance/Personal Independence Payment): https://www.gov.uk/browse/disabilities/benefits

	• Access to Work information including contact details for all centres (for registration, the initial step for clients wanting to use this scheme): https://www.gov.uk/access-to-work/overview<br>• Benefits and Work website offers advice to people re benefits. Some free information, fee for access to additional support http://www.benefitsandwork.co.uk/<br><br>**Debt issues**<br>• https://www.citizensadvice.org.uk/debt-and-money/<br>• https://www.nationaldebtline.org/<br>• http://www.debtadvicefoundation.org/<br><br>**Equipment advice:**<br>• A huge range of IT accessibility info, assessments, resources: http://www.abilitynet.org.uk/<br>• Disabled Living Foundation: http://ww.dlf.org.uk<br><br>**Guidelines:**<br>• Vocational Rehabilitaiton Associaiton Guidelines- free to download upon registration: https://vrassociationuk.com/<br>• BSRM Publications free to download- VR and long term conditions; VR Interagency guidelines:<br>• https://www.bsrm.org.uk/publications/publications<br><br>**Fit Note**<br>• AHP Fitness to Work Report info: http://www.ahpf.org.uk/AHP_Advisory_Fitness_for_Work_Report.htm<br>• Fit Note info: https://www.gov.uk/government/collections/fit-note<br><br>• Managing sickness absence, disputes and sick pay<br>• Gov.uk - https://www.gov.uk/employers-sick-pay<br><br>**The Health and Safety Executive** has provided guidance for employers and managers on managing sickness absence and return to work.<br>• www.hse.gov.uk/pubns/priced/hsg249.pdf<br><br>**British Occupational Health Research Foundation** has also developed guidance for managing sickness absence and return to work. www.bohrf.org.uk/downloads/Managing_Rehabilitation-Guidance.pdf<br><br>For questions about **Statutory Sick Pay** you can visit the HMRC website at https://www.gov.uk/topic/business-tax/paye or call them on 08457 143143.<br><br>**The Employer's Charter** helps employers understand what they can do in respect of a number of issues. www.gov.uk/government/uploads/system/uploads/attachment_data/file/32147/employerscharter.pdf<br><br>• Touchbase: DWP news about work, working age benefits, pensions and services (DWP, 2015) Accessible via: Touchbase: DWP news about work, working age benefits, pensions and services - GOV.UK (www.gov.uk)<br><br>**Job search:**<br>• https://www.gov.uk/jobsearch<br>• http://www.indeed.co.uk<br>• https://jobs.civilservice.gov.uk/company/nghr/jobs.cgi<br>• http://jobs.theguardian.com/<br>• http://www.jobs.nhs.uk/<br>• http://www.charityjob.co.uk/<br>• http://www.jobhuntersbible.com/<br>• http://www.jobsgopublic.com/searches/new

	**Stroke information**<br>• Different strokes - https://differentstrokes.co.uk/ (for younger stroke pts)<br>• Stroke association https://www.stroke.org.uk<br><br>• **VR general:**<br>• MS Trust/Society and Headway - links to toolkits<br>• Job Accommodation Network https://askjan.org/<br>• British Association of Supported Employment http://base-uk.org/<br><br>• **Volunteering associations**<br>• https://www.ncvo.org.uk/ncvo-volunteering<br>• https://do-it.org/<br><br>**Fitness/health information** http://www.nhs.uk/Livewell/fitness/Pages/free-fitness.aspx<br>• Cinema Exhibitor card https://www.cinemauk.org.uk/key-issues/disability-and-access/cea-card/<br>• If a person gets DLA, PIP or is registered blind, they can get this card and it entitles a free entry for another person<br>• Local walk for health schemes http://www.walkingforhealth.org.uk/walkfinder/ -<br><br>**Transport**<br>• DVLA (driver vehicle licencing authority)<br>• https://www.gov.uk/stroke-and-driving (patient information)<br>• https://www.gov.uk/current-medical-guidelines-dvla-guidance-for-professionals<br><br>**Disabled bus pass**<br>• If not allowed to drive for a year due to their injury, they are entitled to a disabled bus pass<br>• https://www.gov.uk/apply-for-disabled-bus-pass<br><br>• **Goal Attainment Scaling (GAS)** in Rehabilitation system https://www.kcl.ac.uk/cicelysaunders/resources/tools/gas<br><br>**Procedures:**<br><br>***Intervention Delivery***<br>ESSVR is an early, individually tailored, stroke specific job retention intervention. It adopts a problem- solving process, which involves vocational goal setting and regular progress review. It aims to adapt the environment and accommodate the stroke survivor at work. It also aims to educate the person to self-manage the condition at work.<br>It involves a trained vocational rehabilitation OT adopting a role as a case coordinator with a wider team of healthcare professionals, employers, family members and other agencies (e.g. occupational health and employment services, GPs, independent and voluntary sector services) to:<br><br>• Assess the impact of the stroke on the patient, family and the patient's role as a worker/student and their ability to do their job/study course.<br>• Educate participants, employers/tutors and families about the effects of stroke and its impact on work/education and find acceptable strategies to lessen the impact.<br>• Monitor and assess the patient's work/educational goals.<br>• Prepare people for work/education by establishing structured routines with gradually increased activity levels and opportunity to practice work skills, e.g., structured computerised cognitive stimulation to increase concentration, daily walks to increase physical stamina.<br>• Liaise with employers/tutors, employment advisors, student services and the healthcare team to advise about the effects of stroke and to plan and monitor a phased return to work.<br>• Alternatives to pre-injury employment are explored in cases where return to pre-existing employer is not feasible or unsustainable.

	The Occupational Therapist VR role involves, negotiating workplace accommodations, communicating with employers, offering advice and emotional to the patient, the patient's family and employer, and exploring work alternatives as required. The case-coordination role involves theRETAKE OT actively coordinating the RTW and input from relevant services from across all sectors (health, work, independent, voluntary, education), communicating with all involved stakeholders, such as the participants GP Department for Work and Pensions Services, welfare rights and employer organisations e.g. occupational health, GPs and voluntary sector services e.g. the stroke Association. The aim being to maximise the use of all locally available resources and ensure consistent advice and support for the patient.<br><br>ESSVR is a process (rather than a set of predetermined components) that is broken into 3 stages;<br><br>**Stage 1: Early recovery and Work preparation:** The OT intervenes early, within 8 weeks of stroke onset, to ensure work is on the agenda and jobs are not relinquished but kept open. Assessment of the individual, the impact of the stroke and a detailed job analysis and liaison with family members takes place at this stage. Plans are made to prepare the RETAKE participant for work return by providing advice and information to the participant and their family and advise medical/other rehab staff to encourage the participant not to make immediate decisions about work i.e. leaving work or going back too soon, which may jeopardise their RTW or job retention. The RETAKE participant is encouraged to keep the channels of communication with the workplace open and the RETAKE OT offers to mediate if difficulties arise. Activities are undertaken at home, relevant to work or simulated to build up the stamina and skills required to return to specific work tasks or roles. These include physical, cognitive or communication based activities depending on how the stroke has affected the RETAKE participant and the demands of their job. Liaison with any other services the person is receiving takes place to ensure there is no overlap and the approach to VR is smoothly coordinated.<br><br>**Stage 2: Graded return to work:** This involves planning, negotiating and implementing a phased return to work (RTW). This might involve a worksite visit, negotiation of realistic timing and identification of workplace adjustments/accommodations to optimise RTW. Liaison with Human Resources (HR), occupational health, other employer bodies and medical teams may also take place. Information and education is provided for employers to increase their understanding of the impact of the stroke on the RETAKE participant and how this might influence their ability to meet job demands. The participant receives feedback on their work performance during this stage. This may involve regular reviews, feedback on progress and supporting the employer to provide feedback on work performance, and the implementation of any modifications to the RTW plan or work role.<br><br>**Stage 3: Job Retention:** This involves monitoring the participant's RTW to ensure work stability and troubleshooting issues that may arise with all stakeholders (patient, employer, family, others) and gradually withdrawing support when the work situation is stable. However, participants and employers can re-access this support as required up to 12 months post randomisation. In some cases where work cannot be sustained or is unfeasible, work alternatives e.g. voluntary work, changes in job type, career are explored. In some cases the intervention may involve supporting retirement or medical withdrawal from work.<br><br>The intervention is delivered in addition to the stroke participant's usual stroke rehabilitation. This will vary depending on local provision and individual participants' needs. Therefore, the RETAKE OT liaises with health care professionals providing usual stroke rehabilitation to clarify and agree roles and ensure that any vocational rehabilitation is provided by the RETAKE OT.<br><br>The RETAKE OT works in partnership with other health, social care, charitable, employment and independent sector service providers in delivering the ESSVR. Any parallel rehabilitation or other wider services involved (e.g. other OTs, Social Services, Jobcentre Plus, Occupational Health, Different Strokes) are kept informed of the ESSVR process, the RETAKE participant's progress and the RETAKE OTs involvement. RETAKE OTs will refer to, liaise with and help participants to access any service they need, and attend DWP appointments or Occupational Health meetings with participants if required.<br><br>Assessment of the impact of the stroke on the person and the job may involve the use standardised assessments of function and impairment e.g. mobility and cognition, functional capacity evaluation, work needs, and detailed job analysis. Specific tools are not prescribed but rather introduced and resources signposted.

	For more detailed descriptions of the intervention delivered in the feasibility trial see;<br><br>Grant M. (2016) Developing, delivering and evaluating stroke specific vocational rehabilitation: A feasibility randomised controlled trial (Doctoral dissertation, University of Nottingham).<br><br>Grant M, Radford K, Sinclair E, Walker M (2014) Return to work after stroke: recording, measuring, and describing occupational therapy intervention. British Journal of Occupational Therapy, 77(9), 457–465.
**WHO PROVIDED** For each category of intervention provider (e.g. psychologist, nursing assistant), describe their expertise, background and any specific training given.	**Intervention provider qualifications**<br>The intervention was delivered by qualified and HealthCare Professions Council (HCPC) registered occupational therapists (OTs).<br><br>**Intervention provider background and experience**<br>The OTs require experience of working with people with stroke and/or other neurological conditions and community rehabilitation experience. Some may have vocational rehabilitation experience.<br><br>The level of experience and suitability of the therapists recruited to deliver the intervention is assessed by the Chief Investigator and OT mentors prior to training.<br><br>**Training provided**<br>The training comprised 2-days of face-to face teaching delivered by the RETAKE training team (4 OTs experienced in vocational rehabilitation and research) followed by an additional day, 6 months later, supported by monthly small group-based (4-6 OTs) telephone/ videocall mentoring from occupational therapists with extensive experience in delivering vocational rehabilitation following stroke. The OT mentors were members of the training team. Three members of the OT training team held a PhD.<br>The purpose of mentoring is to ensure implementation and fidelity to the intervention process through discussion of difficulties and sharing of best practice with other OTs and their mentor.<br><br>Prior to training, occupational therapists were signposted to papers relating to the RETAKE feasibility trial findings and were sent a RTW case study, which required them to provide written responses to 6 questions and return to the training team prior to training. This enabled the expert trainers to ascertain the OTs pre-training vocational rehabilitation knowledge. The same case study was used to teach the ESSVR process during the training.
**HOW**	**Mode of delivery**<br>The intervention is delivered face-to-face or via telerehabilitation (video call or phone call) on a 1 to 1 basis.<br><br>**Other**<br>Additional time is spent in liaison (letters, phone and video calls) with the patient, employer, family or other stakeholders. Most progress monitoring in stage 3 is delivered by telephone.
**WHERE**	**Where provided**<br>The intervention is delivered in the community (mostly in the home or in the workplace). Other locations may include the meeting room of a disability rights charity (13%), and a voluntary organization jobs brokerage centre (7%). In the feasibility trial almost half of the participants were initially seen in hospital or in a stroke rehabilitation unit.

**WHEN and HOW MUCH**.	**Intervention delivery time**<br>The intervention commences within 8 weeks of stroke and continues for up to 12 months following the initial session. The duration of intervention and frequency of contacts is determined by individual participant's needs. Based on feasibility trial data (Grant, 2014), two thirds of the OTS time will be spent delivering the intervention either face-to-face or in liaison with the participant and others. The other third is spent writing notes and reports or travelling to see participants at home or their work places.<br><br>**Number of sessions and length**<br>Based on feasibility trial data the estimated mean number of face-to face sessions per participant is 10 (SD 7, range 1–25) and average session length is one hour. People with more moderate and severe stroke may require more sessions.<br><br>**Frequency of sessions**<br>More interventions sessions will be delivered at the outset of the intervention during stages 1 and 2 with less frequent interventions in stage 3, during progress monitoring once the participant has RTW.
**TAILORING**<br>If the intervention was planned to be personalised, titrated or adapted, then describe what, why, when, and how.	The ESSVR intervention will be tailored in duration and frequency according to individual need over a 12-month period.
**MODIFICATIONS**	During the current trial intervention delivery continued according to local NHS Trust protocols throughout the COVID-19 pandemic. In some sites OTs continued to visit participants at home wearing personal protective equipment, in others delivery was via telerehabilitation (online or telephone).
**HOW WELL**	**Planned**<br><br>Throughout the trial fidelity to the intervention process will be measured and monitored as described in Table 2 and summarised below.<br><br>Frequency duration and dose will be recorded using case report forms (CRFs), capturing Intervention start date and end date, Number of proposed and attended sessions, Whether there was an agreed ending for OT return to work support; Time spent (in minutes) on VR activities per session and from the description of intervention delivered in OT clinical records.<br><br>Adherence and Factors affecting adherence will be measured using an ESSVR fidelity checklist (Powers, in preparation) and recorded on mentoring CRFs during monthly mentoring sessions led by an experienced vocational rehabilitation OT. implementation barriers and contamination risks will be communicated to the trial team, enabling barriers to be managed in real time.<br><br>Factors affecting intervention delivery will be recorded in Interviews with RETAKE Therapists, participants with stroke, their employers and other NHS staff as part of a series of embedded case studies.
Actual: If intervention adherence or fidelity was assessed,	

describe the extent to which the intervention was delivered as planned.	

References

1. Stroke statistics: sources and definitions | Stroke Association 2021, Accessed 18 September 2021
2. Tyerman A, Meehan M. Vocational assessment and rehabilitation after acquired brain injury: inter-agency guidelines. Royal College of Physicians of London. 2004. https://www.bsrm.org.uk/downloads/vocational-assessment-rehabilitation-abi.pdf. Accessed 06 April 2020.
3. Department of Health (DH). National Stroke Strategy. DH. 2007. https://www.england.nhs.uk/south/wp-content/uploads/sites/6/2017/07/national-stroke-strategy-2007.pdf. Accessed 06 April 2002.
4. National Institute for Health and Care Excellence (NICE). Stroke rehabilitation in adults: Clinical guideline [CG162]. 2013. https://www.nice.org.uk/guidance/CG162. Accessed 06 April 2020.
5. NHS England, National Stroke Service Model, National Stroke Programme, 2021 national-stroke-service-model-integrated-stroke-delivery-networks-may-2021.pdf (england.nhs.uk) Accessed 18 September 2021
6. NHS England. NHS Five Year Forward View: NHS England. 2014. https://www.england.nhs.uk/wp-content/uploads/2014/10/5yfv-web.pdf. Accessed 06 April 2020.
7. Intercollegiate Stroke Working Party. National clinical guideline for stroke. 4th ed. Royal College of Physicians. 2012. https://www.strokeaudit.org/Guideline/Historical-Guideline/National-Clinical-Guidelines-for-Stroke-fourth-edi.aspx. Accessed 27 May 2020.
8. Department for Work & Pensions and Department of Health (2017) Improving lives. The future of work, health and disability. London: Stationery Office. Available at: Improving Lives The Future of Work, Health and Disability (publishing.service.gov.uk) Accessed 18 September 2021.
9. Black CM, Frost D. Health at work - an independent review of sickness absence. Department for Work and Pensions. 2011. https://assets.publishing.service.gov.uk/government/uploads/system/uploads/attachment_data/file/181060/health-at-work.pdf. Accessed 06 April 2020.
10. Sinclair E, Radford K, Grant M, et al. Developing stroke-specific vocational rehabilitation: a soft systems analysis of current service provision. Disability and Rehabilitation; 2014; 36:409-17.
11. Royal College of Physicians, Sentinel Stroke National Audit Programme (SSNAP). Post-acute audit. In: Intercollegiate Stroke Working Party (ed.) 2015 Accessible at Sentinel Stroke National Audit Programme (SSNAP) | RCP London, Accessed 18 September 2021
12. Lock S, Jordan L, Bryan K, Maxim J. Work after stroke: focusing on barriers and enablers. Disability & Society. 2005; 20: 33–47
13. Royal College of Occupational Therapists (RCOT). Getting my life back: occupational therapy promoting mental health and wellbeing in England. RCOT. 2018. https://www.kmpt.nhs.uk/media/1059/getting-my-life-back_england.pdf. Accessed 12 May 2020.
14. Public Health England (PHE). Health matters: health and work. PHE. 2019. https://www.gov.uk/government/publications/health-matters-health-and-work/health-matters-health-and-work. Accessed 12 May 2020.Westerlind etc
15. NHS Outcomes Framework (NHS OF) - NHS Digital Summary Tables: NHS Digital. 2021. https://files.digital.nhs.uk/satatistical/nhs-outcomes-framwork. Accessed 18 September 2021.
16. Department for Work and Pensions (DWP) (2020). The employment of disabled people 2019. Available at: https://www.gov.uk/government/statistics/the-employment-of-disabled-people-2019, DWP 2020Accessed 18 September 2021
17. The Equality Act, (2010) accessible at; https://www.legislation.gov.uk/ukpga/2010/15/contents
18. Grant M. Developing, delivering and evaluating stroke specific vocational rehabilitation: a feasibility randomised controlled trial [PhD thesis on the Internet]. Nottingham, UK: University of Nottingham Repository. 2016. http://eprints.nottingham.ac.uk/35108/. Accessed 27 May 2020.

19. World Health Organisation, The International Classification of Functioning Disability and Health. 2001 Available at: International Classification of Functioning, Disability and Health (ICF) (who.int) (accessed 18 September 2021
20. Loisel P, Durand M-J, Berthelette D, et al. Disability prevention: New paradigm for the management of occupational back pain. Disease Management and Health Outcomes, 2001, 9(7): 351–360.
21. Fadyl J, McPherson KM, Schulter P, Turner-Stokes L. Development of a new tool to evaluate work support needs and guide vocational rehabilitation: The Work-ability Support Scale (WSS), Disability and Rehabilitation. 2014. 37:3, 247-258, DOI: 10.3109/09638288.2014.914586
22. Powers K, Clarke S, Cripps R, Holmes JA, Phillips J, Craven K, Farrin A, das Nair R, Radford KA, Developing and testing an implementation fidelity checklist for a complex vocational rehabilitation intervention, in preparation
