# Supplementary material for: RETurn to work After stroKE (RETAKE) Trial: protocol for a mixed-methods process evaluation using normalisation process theory
Source: BMJ Open. 2022 Mar 15;12(3):e053111. doi: 10.1136/bmjopen-2021-053111 (PMC8928258; doi:10.1136/bmjopen-2021-053111)
Supplement: Supplementary data [file bmjopen-2021-053111supp002.pdf]

Appendix II RETAKE OT Competency Marking Rubric

Criteria	**Needs support**	**Competent**	**Highly competent**
	≤49%<br><br>Demonstrates some understanding of ESSVR and its application in RETAKE. However, major deficits noted in VR knowledge, clinical reasoning and application. Requires additional individualised mentoring until next assessment.	50-69%<br><br>Understands ESSVR with some evidence of misinterpretation in its application in RETAKE. Ad hoc monitoring via group mentoring until next assessment.	≥70%<br><br>Fully understands ESSVR and its application in RETAKE.
**Knowledge of intervention processes, timeframes & documentation**<br><br>**(40% of total marks)**	Most answers were missing the required ESSVR components.	Some answers were missing the required ESSVR components.	Few, if any of the required ESSVR components were missing in the answers.
**Clinical reasoning – identification and analysis of salient work-related issues in the case study, to inform the design of an appropriate intervention (ESSVR) plan in the letter/report.**<br><br>**(50% of total marks)**	Limited identification of and/or limited analysis of work-related issues from the case study. None or few solutions for the work-related issues identified within the intervention plan(s). Significant gaps remain in problem-solving.	Some identification of and/or some analysis of work-related issues from the case study. A number of solutions for the work-related issues identified within the intervention plan(s) but a few gaps remain in problem-solving.	Identification and or analysis of all work-related issues from the case study. Comprehensive solutions for the work-related issues within the intervention plan(s).
**Written communication of work issues. Appropriate use of lay language in letter/report to ensure if it is fit for purpose & likely to gain reader engagement.**<br><br>**(10% of total marks)**	Letter/report lacks logical structure. Limited focus of work issue(s) addressed. Overuse of medical terminology. Little use of lay language to communicate issues. Information conveyed in a manner less likely to engage recipient.	Case study letter/report reasonably well structured. Mostly focussed on the work issue(s) being addressed. Minimal use of medical terminology. Good use of lay language to communicate issues. Information conveyed in a manner may to engage recipient.	Case study letter/report very well structured. Report fully focussed on work issue(s) addressed. Issues communicated clearly in lay language and without any use of medical terminology. Information conveyed in a manner likely to engage recipient.

RETAKE OT competency rubric Appendix II
