# Supplementary material for: RETurn to work After stroKE (RETAKE) Trial: protocol for a mixed-methods process evaluation using normalisation process theory
Source: BMJ Open. 2022 Mar 15;12(3):e053111. doi: 10.1136/bmjopen-2021-053111 (PMC8928258; doi:10.1136/bmjopen-2021-053111)
Supplement: Author's manuscript [file bmjopen-2021-053111.draft_revisions.pdf]

BMJ Open

BMJ Open is committed to open peer review. As part of this commitment we make the peer review history of every article we publish publicly available.

When an article is published we post the peer reviewers' comments and the authors' responses online. We also post the versions of the paper that were used during peer review. These are the versions that the peer review comments apply to.

The versions of the paper that follow are the versions that were submitted during the peer review process. They are not the versions of record or the final published versions. They should not be cited or distributed as the published version of this manuscript.

BMJ Open is an open access journal and the full, final, typeset and author-corrected version of record of the manuscript is available on our site with no access controls, subscription charges or pay-per-view fees (http://bmjopen.bmj.com).

If you have any questions on BMJ Open's open peer review process please email info.bmjopen@bmj.com

BMJ Open

The RETurn to work After stroKE (RETAKE) Trial: protocol for a mixed-methods process evaluation using normalisation process theory

Journal:	*BMJ Open*
Manuscript ID	bmjopen-2021-053111
Article Type:	Protocol
Date Submitted by the Author:	05-May-2021
Complete List of Authors:	Radford, Kathryn A; University of Nottingham<br>McKevitt, Christopher; King's College London, Department of Public Health Sciences<br>Clarke, Sara; University of Nottingham, Division of Rehabilitation, Aging and Wellbeing<br>Powers, Katie; University of Nottingham<br>Phillips, Julie; University of Nottingham<br>Craven, Kristelle; University of Nottingham<br>Watkins, Caroline; University of Central Lancashire, Faculty of Health and Wellbeing<br>Farrin, Amanda; University of Leeds, Clinical Trials Research Unit<br>Holmes, Jain; University of Nottingham<br>Cripps, Rachel; King's College London<br>McLellan, Vicki; University of Leeds, Clinical Trials Unit<br>Sach, Tracey; University of East Anglia, School of Chemical Sciences and Pharmacy<br>Brindle, Richard; University of Leeds, Clinical Trials Unit<br>Holloway, Ivana; University of Leeds, Clinical Trials Unit<br>Hartley, Suzanne; University of Leeds, Clinical Trials Research Unit<br>Bowen, Audrey; The University of Manchester, DNEP, SBS, FBMH<br>O'Connor, Rory J.; University of Leeds<br>Stevens, Judith<br>Walker, Marion; University of Nottingham<br>Murray, John<br>Shone, Angela; University of Nottingham<br>Clarke, David; University of Leeds, Academic Unit of Elderly Care and Rehabilitation
Keywords:	Stroke < NEUROLOGY, Clinical trials < THERAPEUTICS, STROKE MEDICINE

SCHOLARONE™
Manuscripts

TITLE

The RETurn to work After stroKE (RETAKE) Trial: protocol for a mixed-methods process evaluation using normalisation process theory

NAMES OF PROTOCOL CONTRIBUTORS

Kathryn A Radford[1*], Christopher McKevitt[9], Sara Clarke, Katie Powers, Julie Phillips[1], Kristelle Craven[1], Caroline Watkins[4], Amanda Farrin[2] , Jain Holmes[1], Rachel Cripps[9], Vicki McLellan[2], Tracey H Sach[3], Richard Brindle[2], Ivana Holloway[2], Suzanne Hartley[2], Audrey Bowen[8], Rory O'Connor[5], Judith Stevens[6], Marion Walker[1], John Murray[7], Angela Shone[10], David J Clarke[11]

*Correspondence: Kathryn.radford@nottingham.ac.uk

[1]Division of Rehabilitation, Ageing and wellbeing, School of Medicine, B-Floor, Medical School Queen's Medical Centre, Nottingham NG7 2UH. Full details of authors' information are presented at the end of the article.

WORD COUNT: 3965 (minus abstract, strengths and limitations, tables/figures and references)

For peer review only

For peer review only - http://bmjopen.bmj.com/site/about/guidelines.xhtml

ABSTRACT

Objectives

This mixed-method process evaluation underpinned by Normalisation Process Theory (NPT) aims to measure fidelity to the intervention, understand the social and structural context in which the intervention is delivered, and to identify barriers and facilitators to intervention implementation.

Setting

Return to Work after Stroke (RETAKE) is a multi-centre individual patient randomised controlled trial to determine whether Early Stroke Specialist Vocational Rehabilitation (ESSVR) plus usual care is a clinically and cost-effective therapy to help people return to work after stroke, when compared with usual care alone. This protocol paper describes the embedded process evaluation.

Participants and outcome measures

Intervention training for therapists will be observed and use of remote mentor support reviewed through documentary analysis. Fidelity will be assessed through participant questionnaires and analysis of therapy records, examining frequency, length and content of ESSVR sessions. Therapists' attitudes towards evidence-based practice, their competency to deliver the intervention and identification of potential sources of contamination will also be evaluated. Longitudinal case studies incorporating non-participant observations will be conducted with a proportion of intervention and usual care participants. Semi-structured interviews will be completed with stroke survivors, carers, occupational therapists, mentors, service managers and employers. Analysis of qualitative data will draw on thematic and

For peer review only - http://bmjopen.bmj.com/site/about/guidelines.xhtml

Framework approaches. Analysis of quantitative data focused on intervention fidelity will include regression models and descriptive statistics.

Conclusions

Large trials of complex rehabilitation interventions often lack empirical data needed to provide context for interpreting trial outcomes. Embedded process evaluations are vital to understanding factors impacting on, and potentially influencing, trial results. The process evaluation will also identify professional and organisational implications of embedding and sustaining an ESSVR intervention in post-stroke rehabilitation services.

Trial registration

Registration number: ISRCTN: 12464275

KEYWORDS

Return to work, stroke, vocational rehabilitation, occupational therapy, complex intervention, process evaluation, randomised controlled trial, mixed-methods, qualitative, Normalisation Process Theory, Consolidated Framework for Implementation Fidelity

STRENGTHS AND LIMITATIONS OF THIS STUDY

- A mixed-methods theory-driven process evaluation will generate detailed findings to assist in interpreting the results of a pragmatic, multi-centre

For peer review only - http://bmjopen.bmj.com/site/about/guidelines.xhtml

individual patient randomised controlled trial of a complex vocational rehabilitation intervention, which crosses the work/health divide.

- This is one of the most comprehensive multi-site, multi-component, multi-stakeholder perspective process evaluations embedded in a stroke rehabilitation trial, involving detailed assessment of implementation fidelity, therapist competency to deliver the trial intervention, contamination logging and exploration of social and structural influences on intervention provision in post-stroke rehabilitation services.
- Longitudinal case studies with intervention and usual care will capture participant experiences of providing and experiencing the intervention including those of employers.

BACKGROUND

Approximately 100,000 people in the UK suffer from a stroke every year,[1] and around 1 in 4 are of working age.[2] Returning to work after a stroke is a major goal for stroke survivors, contributing to social identity, emotional and financial wellbeing, and conferring a sense of purpose and has benefits for the individual, the individual's family and the economy.[3] Despite this, only half of working age stroke survivors make a successful return to meaningful work, and they are two to three times more likely to be unemployed eight years after their stroke than the general population.[1] Although impairments in the stroke survivor's physical, cognitive and communication abilities can affect this,[4, 5] social and environmental factors such as personal and

For peer review only - http://bmjopen.bmj.com/site/about/guidelines.xhtml

employer beliefs and attitudes, job type and organisation size and the benefits system also play an important part.[6, 7]

Vocational rehabilitation (VR) is defined as whatever helps someone with a health problem to return to, or remain in, work and includes both work *and* work-related education.[8] It involves helping people find work, helping those who are in work but having difficulty, as well as supporting career progression in spite of illness or disability. The primary aim is to optimise work participation.[9] Existing research suggests that VR may help stroke survivors return to their previous job or find new work,[10, 11] however trials to date involve small samples in non-UK settings.

RETAKE is a multi-centre individual patient randomised controlled trial (RCT) which aims to determine the clinical and cost-effectiveness of an Early Stroke Specialist Vocational Rehabilitation (ESSVR) intervention in addition to usual NHS rehabilitation on stroke survivors' return to work at 12 months post-randomisation, compared to NHS rehabilitation alone.[12] Acceptability and utility were assessed in a feasibility trial.[13] ESSVR combines conventional occupational therapy (OT) with case coordination and is intended for delivery in the community as often as required by individuals, as determined by a stroke specialist OT with additional VR training. ESSVR includes the following: (a) assessing stroke impact on the person and their job; (b) educating individuals, employers, and families about stroke impact on work, and strategies to lessen impact (e.g. memory aids, fatigue management); (c) work preparation, including opportunities to practice work skills; and (d) liaison with employers to plan and monitor a phased return to work (RTW).

For peer review only - http://bmjopen.bmj.com/site/about/guidelines.xhtml

Failure to implement evidence-based stroke rehabilitation interventions in clinical practice may result in unnecessary suffering and disability.[14, 15] Trialists must consider future implementation in the real world when designing clinical trials, paying particular attention to the context for intervention delivery and factors likely to influence its uptake and use.[16] This is especially true for trials of complex rehabilitation interventions, which comprise multiple interacting components, and target a number of different organisational levels, making them particularly challenging to implement. An embedded process evaluation provides for an in-depth exploration of factors influencing the implementation of complex interventions.

The Medical Research Council (MRC) argue for a systematic approach to designing and conducting process evaluations, drawing on clear descriptions of intervention theory and the identification of key process questions.[17] Mixed-method approaches to process evaluation are increasingly common and consistent with the MRC framework's emphasis on exploring and understanding the important relationship between context, mechanisms and implementation. Theory driven process evaluations are recommended alongside complex intervention trials to measure what is delivered. These measurements include fidelity (whether the intervention was delivered as intended), dose (the quantity of intervention implemented), and "reach" of interventions to understand how the intended audience interacts with the intervention.[17] Alongside a focus on fidelity, in-depth qualitative exploration of participants' experiences of an intervention, and of the social and structural context in which an intervention is provided, are essential elements of process evaluation of complex interventions. This ensures any adaptations made to tailor intervention to the individual and/or differing contexts, which might undermine fidelity can be

For peer review only - http://bmjopen.bmj.com/site/about/guidelines.xhtml

evaluated. Understanding and reporting how the intervention (including training and support, communication and management structures) is delivered is important for replication in clinical practice.[17] Such evaluation aims to reduce the chance of discounting effective interventions (Type II error) or erroneously attributing outcomes to treatment effectiveness, when interventions are not delivered as intended (Type III Errors).[18 - 21] The approach is designed to improve trial design and knowledge translation interventions enhancing clinical implementation and reducing research waste.[22, 23]

This paper reports the protocol for the process evaluation embedded in the RETAKE trial.

AIMS AND OBJECTIVES

Aims

To determine OTs competency to deliver the ESSVR intervention, measure fidelity to the ESSVR intervention and understand the social and structural context in which the intervention is delivered and identify factors which may influence the quality of implementation.

Objectives

Fidelity measurement and competency assessment will

1. Ascertain intervention dose

For peer review only - http://bmjopen.bmj.com/site/about/guidelines.xhtml

2. Describe content of usual care and ESSVR

3. Describe levels of adherence to the ESSVR intervention

4. Understand the delivery of Usual Care and ESSVR.

5. Determine OTs competency to deliver ESSVR

Social and structural context will include

6. Describe participating sites.

7. Understand professionals' experiences of being trained to deliver the intervention.

8. Understand experiences of delivering the intervention.

9. Understand the social and structural factors which support the implementation of the intervention.

10. Understand participants' experience of being supported to return to work after stroke.

11. Identify potential contaminants

METHODS

Design

Embedded theory-driven mixed-methods process evaluation incorporating qualitative and quantitative methods. The process evaluation will draw on the intervention logic model developed by the Trialists (Figure 1) and will be underpinned by Normalisation Process Theory (NPT), an implementation theory built on four constructs (coherence, cognitive participation, collective action and reflexive monitoring) each informed by four components.[24] NPT will be used in the development of data

For peer review only - http://bmjopen.bmj.com/site/about/guidelines.xhtml

collection tools (interview topic guides and observation checklists) and as a sensitising lens in qualitative data analysis and interpretation. NPT constructs will underpin the process evaluation and provide insights into the implementation and integration of the intervention into participating stroke services. This will include how the intervention is received, understood, implemented and how it could be normalised into the current healthcare system (see Table 1).

Figure 1. The ESSVR logic model.

Table 1: Normalisation Process Theory (Adapted from May et al, 2015)

NPT constructs	Components	Explanation
**Coherence**	• Differentiation<br>• Communal specification<br>• Individual specification<br>• Internalisation	The sense making work that people do individually and collectively when faced with implementing changes to existing working practices. This would include differentiating new practices from existing work and thinking through not only the perceived value and benefits of desired/planned changes but also what work will be required of individual people in a setting to bring about these changes.
**Cognitive Participation**	• Initiation<br>• Enrolment<br>• Legitimation<br>• Activation	The work that people need to do to engage with and commit to a new set of working practices. This often requires bringing together those who believe in and are committed to making changes happen. This also involves people working together to define ways to implement and sustain the new working practices.

For peer review only - http://bmjopen.bmj.com/site/about/guidelines.xhtml

**Collective Action**	• Interactional workability<br>• Relational integration<br>• Skill set workability<br>• Contextual integration	The work that will be required of people to actually implement changes in practices, including preparation and/or training of staff. Often this entails rethinking how far existing work practices and the division of labour in a setting will have to be changed or adapted to implement the new practices. This requires consideration of not only who will do the work required, but also the skills and knowledge of people who will do the work and the availability of the resources they need to enact and sustain the new working practices.
**Reflexive monitoring**	• Systematisation<br>• Communal appraisal<br>• Individual appraisal<br>• Reconfiguration	Peoples' individual and collective on-going informal and formal appraisal of the usefulness or effectiveness of changes in working practices. This involves considering how the new practices affect the other work required of individuals and groups and whether the intended benefits of the new working practices are evident for the intended recipients and staff.

In addition, the Conceptual Framework for Implementation Fidelity (CFIF) (Figure 2) will guide collection and analysis of quantitative data.[25] The CFIF outlines the components and variables that make up and affect intervention fidelity and explains how they relate to each other. Adherence includes content and dose (frequency, coverage and duration) of the delivery.[25]

Figure 2. Assessment of fidelity and factors moderating ESSVR delivery in accordance with the Conceptual Framework for Implementation Fidelity.[25]

Eligibility criteria

For peer review only - http://bmjopen.bmj.com/site/about/guidelines.xhtml

Stroke survivors that meet the following criteria will be considered eligible to participate in the process evaluation:

- Age ≥18 years.
- Admitted to hospital with new stroke (all severities).
- In work at stroke onset (including self-employed, paid or voluntary).
- Willing and have capacity to provide informed consent to participate in the study.
- Have sufficient proficiency in English to contribute to the data collection required for research.

Potential participants who do not intend to return to work will be excluded.

Inclusion criteria for carers of potential participants:

- Nominated carer of consenting participant.
- Willing and have capacity to provide informed consent to participate in the study.
- Have sufficient proficiency in English to contribute to the data collection required for research.

Informed Consent

Potential participants will be provided with an information sheet and be provided the opportunity to ask questions of a researcher prior to consent. Written informed consent will be obtained from all participants. When a participant is randomised to the case study element, a researcher will contact the participant to gain consent for interview and observations. Consent will be reaffirmed at the start of interviews. This process will be the same for carer, employer, OT and NHS staff interviews. For

For peer review only - http://bmjopen.bmj.com/site/about/guidelines.xhtml

employer interviews, additional consent to contact the employer will be requested from the case study participant before the employer is contacted.

Patient and Public Involvement Statement

Stroke survivors are involved in all stages of the research cycle.

Design and development.

Two stroke survivors are co-applicants on the grant and assisted in identifying the research questions, designing the study and developing the trial protocol.

Delivery.

Two PPI are members of the Trial Steering Committee, and two are members of the Trial Management Group. Additionally, our RETAKE PPI (Patient & Public Involvement) group, which has six members, meets quarterly. Examples of the work achieved by the PPI group to date are:

- Helping define the primary outcome and defining 'voluntary work' which is included in the definition of the primary outcome.
- Evaluating all patient facing material including aphasia friendly recruitment material.
- Co-development of interview topic guides for trial participants and occupational therapists.
- Overcoming problems with recruitment. For example, resources and narratives to assist recruiters in approaching people with severe stroke.
- Assisting in the design of new materials to promote follow up e.g. including a 'patient journey leaflet' and Thankyou cards.
- Helping reduce the length of follow-up questionnaires.
- Advising on communicating with participants during the pandemic.
- Changes to the Excess Treatment Cost payment models during trial, caused problems for the study. One PPI member wrote directly to Directors of the

For peer review only

NIHR, NHS England, Health and Social Care and the leads for the NIHR Clinical Research Network to explain the impact that these changes on the trial. She received a prompt response which was extremely helpful to the research team. This has assisted us in explaining the new system to clinical colleagues and researchers in the Trusts.

- Co-Development of a trial website and trial newsletters.

The PPI group will also be involved in writing up and presenting study findings.

Data Collection

The process evaluation will employ qualitative and quantitative methods to address the research questions. Table 2 illustrates the relationship between the process evaluation aims, research questions, data sources and data collection methods. The following section describes each data source in more detail.

Table 2: RETAKE process evaluation research questions and data sources

Aims	Research questions	Data Source(s)	Method(s)
**Measure fidelity to the intervention**	What is the intervention dose, intensity and duration?	• Intervention content case report forms (CRFs)	Quantitative
	What is the content of the RETAKE intervention?<br><br>What is the content of usual care?	• Intervention content CRFs.<br>• NHS therapy records.<br>• Stroke survivor-reported resource use data.	Quantitative and qualitative

For peer review only - http://bmjopen.bmj.com/site/about/guidelines.xhtml

		• Stroke survivor carer and OT interviews	
	Was the intervention delivered with fidelity?<br><br>What factors affect implementation fidelity? (context, adherence, moderating factors)	• Fidelity checklist,<br>• Intervention content CRFs<br>• Mentoring records,<br>• RETAKE OT interviews	Quantitative and qualitative
**Determine RETAKE OT competency**	Are the RETAKE OTs competent to deliver the RETAKE intervention?	• Individual OT performance in assessed vignettes at baseline and 6 months<br>• RETAKE OT case records at 12 months post training	Quantitative
**Understand the social and structural context and identify factors which may influence intervention quality (enablers and barriers, contextual factors associated with variations in outcome across the intervention groups, factors supporting**	What is the context for intervention delivery?<br><br>What are the existing stroke pathways?	• Site survey at baseline, mid-point and end of intervention delivery	Quantitative and qualitative
	What services are in place for supporting patients in return to work?	• Site survey at baseline, mid-point and end of intervention delivery	Quantitative and qualitative
	What are the staffing levels at the site?	• Site survey at baseline, mid-point and end of intervention delivery	Quantitative and qualitative

For peer review only - http://bmjopen.bmj.com/site/about/guidelines.xhtml

**implementation into routine practice).**	Are there any proposed VR service developments or changes in place/ planned at site?	• Site survey at baseline, mid-point and end of intervention delivery<br>• NHS staff interviews	Quantitative and qualitative
	What are the RETAKE OTs' perceptions of the training and mentoring to deliver the intervention?	• Observations at training sessions<br>• RETAKE OT interviews	Qualitative
	How do the RETAKE OTs experience delivering the intervention?	• Observations of ESSVR sessions<br>• RETAKE OT interviews<br>• Mentoring records	Qualitative
	What are the social and structural factors supporting intervention implementation?	• Observations of usual care and ESSVR sessions<br>• RETAKE OT interviews<br>• Usual Care therapist interviews<br>• NHS Staff interviews<br>• Mentor interviews	Qualitative
	How do participants' experience being supported to return to work after stroke	• Stroke survivor interviews<br>• Carer interviews<br>• Employer interviews	Qualitative
**Identify potential contaminants.**	What factors threaten the success of the trial?	• Training delivery<br>• Mentoring records<br>• Site survey at baseline, mid-point and end	Quantitative and qualitative

For peer review only - http://bmjopen.bmj.com/site/about/guidelines.xhtml

		of intervention delivery<br>• NHS staff interviews<br>• RETAKE OT interviews<br>• Stroke Participant interviews	

Intervention content Case Report Forms (CRFs)

Initial Session CRFs (one per participant) record the Intervention start date and whether this occurred within 8 weeks of stroke. Participant Summary CRFs record the number of sessions attended out of those proposed and whether there was an agreed ending for the OT led return to work support. To ascertain intervention dose and describe intervention content, data will be extracted from intervention CRFs for all participants (see Table 3). Therapists record each intervention session against pre-defined components, on an 'Intervention content CRF'. These data will be used to identify which components of the intervention were delivered, to what extent therapists adhered to the intervention process described in the RETAKE manual, and to what extent participants adhered to the intervention. For case study participants only, content data will be cross-referenced with the OT's clinical case notes and additional data extracted to explain how the RETAKE intervention interacts with usual care and other services such as employment services.

Describing usual care

To describe the content of the intervention and of usual care, resource use questions pertaining to participants' use of health and social care services over the previous three months will be completed by all participants at three, six and twelve months

For peer review only

For peer review only - http://bmjopen.bmj.com/site/about/guidelines.xhtml

post-randomisation as part of follow-up. This data will be used to describe the content of usual care, and in case study participants (n=38) will be triangulated with therapists' clinical notes and participant interview transcripts.

Therapist competency assessment

Following attendance at a two-day, manualised face-to-face training session with VR expert trainers and again at refresher training six months later, retake OTs competence will be assessed using vignettes depicting novel RTW after stroke scenarios. Model answers developed by the training team will be used to measure competence using criteria based on knowledge of the intervention process (40%), clinical reasoning (50%) and written communication (10%). Scores will be mapped to a rubric identifying OTs as highly competent (≥70%), competent (50-69%) or needing additional support (≤49%). After 12 months of delivering the intervention RETAKE OTs competence will be reassessed by evaluating the intervention delivered in a random selection of completed intervention case records (one participant per RETAKE OT) against the trainer's expert opinion.

Fidelity

To assess implementation fidelity a range of data collection methods informed by the CFIF will be used (see Table 3).[25]

Fidelity Checklist

A fidelity checklist based on the RETAKE intervention logic model (see Figure 1) and RETAKE intervention process and components will be applied to complete case

For peer review only - http://bmjopen.bmj.com/site/about/guidelines.xhtml

315 records (Content of Intervention CRFs, RETAKE OT case notes and Initial Session CRFs) from a random selection of stroke participants randomised to receive the RETAKE intervention (one per treating RETAKE OT). This will be used in measuring adherence to the RETAKE process and identifying factors affecting adherence.

Table 3. CFIF led data extraction for Fidelity Assessment:

Fidelity Measure	CFIF Construct*	Measurement tool	Data for extraction	Time point
Frequency<br><br>Duration	**Adherence and moderating factors**	**Initial Session Case Report Forms (CRFs)**<br><br>**Participant Summary CRFs**	Intervention start date and end date<br>Number of proposed and attended sessions<br>Whether there was an agreed ending for OT return to work support.	One CRF per participant at Initial session.<br><br>One CRF per participant completed throughout intervention delivery
Intensity (time spent per session)<br>Dose (number of sessions)	**Adherence**	**Intervention content CRF**<br><br>**OT clinical records (RETAKE+ Usual Care)**	Time spent (in minutes) on VR activities per session<br><br>Description of intervention delivered in each session	One completed following every intervention session<br><br>In case study participants.
Therapist adherence<br>Factors affecting adherence	**Adherence and moderating factors**	**Fidelity Checklist**	Components delivered, factors affecting delivery<br>RETAKE process followed Y/N	Applied to one randomly selected completed case per RETAKE OT

For peer review only - http://bmjopen.bmj.com/site/about/guidelines.xhtml

Real time therapist adherence<br>Factors affecting adherence	**Adherence and moderating factors**	**Mentoring CRFs**	Mentor's concerns about adherence<br>Factors affecting intervention delivery<br><br>Potential solutions	Completed monthly by mentors
Barriers and enablers to intervention delivery	**Moderating factors**	**Interviews with RETAKE Therapists**	Factors affecting intervention delivery<br><br>Potential solutions (developed by OT)	In a random selection of cases during intervention delivery at 3, 6 and 12 months
Acceptability of the intervention<br>Barriers and enablers to intervention delivery	**Moderating factors**	**Interviews with stroke participants, carers, employers and NHS staff**	Acceptability of intervention<br>Factors affecting delivery<br>Potential solutions to barriers	Throughout intervention delivery in case studies

Key; **CFIF Adherence includes intervention content, dose, coverage, frequency and duration of intervention; CFIF Moderating factors includes participant responsiveness, intervention complexity, strategies to facilitate implementation, quality of delivery, recruitment, and context.*

Mentor interviews and records

Mentoring records

Following training, each treating OT will be assigned a mentor with extensive knowledge and experience of vocational rehabilitation. Mentoring will take place monthly via teleconference in small groups (four to six therapists) and serve as an intervention implementation support mechanism. RETAKE OTs will be able to

For peer review only

For peer review only - http://bmjopen.bmj.com/site/about/guidelines.xhtml

discuss any difficulties they are experiencing, ask questions and share best practice with other OTs and their mentor. This process will also facilitate communication between the trial team and enable barriers to implementation and contamination risks to be reported. Key discussion points will be recorded by mentors using a mentoring record form for each session. These records, along with all email correspondence between mentor and mentees will be collected for qualitative content analysis.

Mentor Interviews

Semi-structured interviews will be conducted by two research assistants (SC and KC) with all mentors (n=6) to explore their experiences of supporting RETAKE OTs to deliver the intervention, and ascertain their views of organisational, social and other factors contributing to or affecting delivery of the intervention.

Social and structural context

Site survey

To describe participating sites and identify potential contaminants, sites will be asked to complete a questionnaire by telephone at three time points; prior to recruitment, halfway through, and at the end of the intervention period. This will contribute to understanding contextual influences through capturing data on existing stroke care pathways and resources (including staff and services) available for supporting participants in a return to work. It will also identify potential contamination risks associated with proposed or planned VR service developments or changes in practice that may influence trial outcomes.

For peer review only - http://bmjopen.bmj.com/site/about/guidelines.xhtml

Therapist training

Non-participant observations

To understand OT's experiences of being trained to deliver the intervention, a research assistant (RC) will observe up to four training sessions delivered by the training team. A checklist will be developed using NPT constructs to guide observations. Non-participant observations aim to identify; whether therapists understand the intervention and their role in implementation, whether they think the RETAKE intervention can be integrated into existing practice and any contextual factors affecting the trial.

To describe adherence to the intervention, a researcher will observe up to three sessions for each case study participant in the intervention and usual care arms of the trial. Non-participant observations will be conducted using prompts for structured observation and unstructured field notes.[26] Participant selection for inclusion the case study element is described below.

Interviews with Occupational Therapists

Semi-structured interviews will be conducted by a research assistant (RC) with a minimum of one OT per site following their initial RETAKE training to explore their experience of training, the mentoring process and their confidence in intervention delivery. OT's views of the intervention, barriers and facilitators to implementation, and any organisational or social factors impacting on delivery will also be explored. Interviews will take place following training and be repeated at two additional time-

For peer review only - http://bmjopen.bmj.com/site/about/guidelines.xhtml

380 points: mid-way through the RETAKE intervention delivery and at the end of the
381 study.
382
383 *Case studies*
384 Longitudinal case studies will be used to map the care received by RETAKE and
385 usual care participants to develop a more detailed understanding of participants'
386 (stroke survivors, carers, employers) and RETAKE OTs experiences of support for
387 RTW. A 5% subset of participants from both arms of the trial (total n=38) will be
388 randomly selected and invited to participate in the case study element of the process
389 evaluation.
390
391 i) Case study interviews
392 Semi-structured interviews will be conducted by two research assistants (SC and
393 KC) with case study participants at three time points: three, six, and twelve months
394 post-randomisation, about their experiences and views of and adherence to the
395 RETAKE intervention and the support they received to return to work. The case
396 study participants' carers (if nominated), their employers (where participant consent
397 is obtained) and the OTs providing support for RTW will be interviewed.

398 NHS staff interviews

399 To further understand the social and structural factors which influence the
400 implementation of the intervention, interviews will be conducted with up to two (n=34
401 in total) NHS staff involved in the management, commissioning or delivery of stroke
402 rehabilitation within each trial site. Participating staff will be chosen using a mixture
403 of purposive and snowball sampling. This will based on a full range of trial sites,

For peer review only - http://bmjopen.bmj.com/site/about/guidelines.xhtml

staff knowledgeable about the implementation of the intervention at their site, and staff knowledgeable about the decision-making process relating to wider roll-out.

Additional participant interviews

An additional random 5% of study participants will be invited to participate in semi-structured interviews at the end of the intervention period. These interviews will explore participants' experience of the intervention as well as their perceptions and experiences of returning to work.

All qualitative interviews will be conducted using a topic guide informed by NPT. Examples of question topics and how they relate to the four NPT constructs are shown in Table 4. Topic guides will be presented to the RETAKE Public and Patient Involvement (PPI) group for comment prior to use. All interviews will be audio recorded and transcribed in full.

Table 4: Examples of question topics related to NPT constructs

**Normalisation Process Theory Constructs**	**NHS Staff/ therapist interview topics** (some may also arise in informal feedback during training observations)	**Stroke Participant interview topics** (some may also arise in intervention / usual care observations)	**Employer interview topics**
**Coherence**	How do staff describe the intervention?<br><br>How is the intervention similar to/different from usual care?<br><br>Who would (most) benefit from the	RTW support received: similarities/differences between control and intervention participants	Experience of liaising with the therapist and/or participant on RTW issues

For peer review only - http://bmjopen.bmj.com/site/about/guidelines.xhtml

	intervention?		
**Cognitive participation**	Do staff see value/potential in the intervention?<br><br>Have they found the training and experience a worthwhile investment of time?<br><br>Do they feel they have the competence/ resources to deliver the intervention effectively?	What were their expectations? Did patients (& carers) value the intervention?<br><br>How did they respond to the therapists' suggestions?<br><br>Did they feel they had the ability/resources/confidence to progress through the sessions and ultimately RTW?<br><br>Context in which participant received RETAKE/acted on suggestions: social, financial, health state, access to opportunities	Expectations of the processes: liaising with therapist/patient and patient's RTW<br><br>(Prior) experience in supporting RTW for people with disabilities
**Collective action**	How compatible is the intervention with the existing stroke care pathway?<br><br>What other RTW services/resources exist locally? How does this intervention compare/complement those services? Describe working relationships with those services.<br><br>Support from managers and colleagues during the intervention period	How did participants accommodate the intervention sessions/follow up actions?<br><br>How did they manage/are they managing their RTW (if applicable)?<br><br>Financial implications	Views on who is responsible /roles in supporting RTW<br><br>Financial implications e.g. modifications
**Reflexive monitoring**	Perceived effects on patients (& carers)<br><br>Views on time/resources invested in delivery vs impact<br><br>What is needed to	Perceived effects of RETAKE/other RTW support<br><br>Views on time/resources invested in participation vs impact<br><br>What was good about	Perceptions of benefit to employer/tutor/advisor<br><br>Perceptions of benefit to employee<br><br>What was helpful about discussions

For peer review only - http://bmjopen.bmj.com/site/about/guidelines.xhtml

	make it possible to roll out the intervention effectively? (changes to intervention; changes in services/resources needed for delivery)	RETAKE and what could be improved? (content of intervention sessions/work plans, timing, relationship with therapist)	with therapist/participant?<br><br>What further information/support would they have liked – at what time?

Data Analysis

Quantitative analysis

The dose, duration and frequency of the ESSVR intervention will be calculated using data from completed CRFs in combination with NHS therapy records. The total time spent delivering the ESSVR intervention (face to face and non-face to face contact (liaison with the patient, employer and other stakeholders by letter/phone), administration and travel) will be identified. Details relating to the content of intervention sessions will be extracted to identify whether core components of ESSVR were delivered as intended (i.e. as specified in the intervention manual and logic model). Associations between therapist attributes, contextual factors and intervention fidelity (measured by deviations from the RETAKE core process) will be explored using regression models. Analysis will be conducted using Statistical Package for the Social Sciences (SPSS) (version 21.0 for Windows). In addition, a fidelity monitoring checklist will be used to check whether the ESSVR process is followed.

Describing Usual Care

Data regarding rehabilitation delivered in Usual Care will be extracted from resource use data in the follow-up questionnaires and from NHS Therapy records in case

For peer review only - http://bmjopen.bmj.com/site/about/guidelines.xhtml

440 study participants randomised to Usual Care. These data will be used to inform the cost of Usual Care for the economic evaluation and describe and understand usual care provided during stroke rehabilitation in inpatient and community services. Quantitative data analysis will be conducted using Statistical Package for the Social Sciences (SPSS; Version 21.0 for Windows). Analysis of usual care data obtained from NHS Therapy records is described below.

Qualitative analysis

Inductive (thematic analysis) and deductive (informed by NPT) approaches will be used guide data analysis and interpretation. Observational and Interview data will be transcribed verbatim and uploaded into QSR NVivo software for management. Descriptions of usual care in NHS Therapy records, observational field note data, including researcher reflections and interview data will be analysed thematically.[26] Framework analysis will be used with the case study data to facilitate within and between case analyses. Analysis of each data set will be conducted independently and then jointly by at least two study team members (SC, KC, KP) to corroborate themes and discuss any discrepancies. It will follow a standard approach of data familiarisation, line-by-line coding, development and refinement of broader conceptual explanatory categories and iterative testing of interpretation through participant feedback and discussions within the research team. Analysis will proceed iteratively with data collection to determine whether data saturation has been achieved; researchers will draw on the RETAKE logic model (Figure 1). Throughout the qualitative analysis, NPT will be used as a sensitising framework. Researchers will keep a set of interim summary notes documenting any reflexivity points and

For peer review only

For peer review only - http://bmjopen.bmj.com/site/about/guidelines.xhtml

connections between the data with NPT and the logic model, to aid analytical discussions with the wider process evaluation team.

DISCUSSION

Process evaluations are increasingly embedded in trials of complex interventions,[16] but published process evaluations of complex stroke rehabilitation trials are still relatively few in number.[29-36] At present, despite the publication of the MRC guidelines for process evaluation,[17] there is limited consensus on how best to conduct these important studies, particularly in relation to complex interventions such as RETAKE, which cross the boundary between health and employment services.

Balancing the need to gain greater understanding of contextual factors that may affect trial outcomes with the realities of collecting more data than is necessary to describe the facilitators and barriers to implementation is a challenge for researchers.[23] However, adopting a robust theoretical framework to underpin the process evaluation, pre-determining objectives that steer the data collection and drawing on previous research mitigates this challenge.[29, 31, 34, 36] Using a mixed-methods approach and generating quantitative data to measure fidelity and adherence to the intervention protocol alongside site specific data and in-depth qualitative data from a wide range of participants will ensure a focused but comprehensive data set to support analysis of the trial outcomes.

The MRC guidelines identify that different approaches to managing process evaluations are used.[17] In this study the process evaluation is led by a researcher

For peer review only - http://bmjopen.bmj.com/site/about/guidelines.xhtml

who is independent of the trial team. However, data will be collected and analysed by researchers who are also contributing to the trial data collection. The development of topic guides and interview schedules with the support of the process evaluation lead has been outlined above. In respect of the qualitative analysis the use of independent and then joint coding and development of themes, followed by review of emerging findings by the wider research team is designed to enhance the transparency and trustworthiness of the analytical process. Research reflexivity is encouraged and recorded in memo form and discussed by the wider research team in process evaluation review meetings every two months.

Rehabilitation interventions are frequently tailored to the participant and modified to suit the local context and resources. It is therefore important to monitor intervention delivery to ensure fidelity is maintained and any moderating factors are identified and addressed in real time to ensure robust trial outcomes. A unique feature of this trial is the use of mentoring for individual RETAKE OTs throughout intervention delivery in this study. Monitoring this process will enable any intervention modifications to be identified and documented in detail. Using NPT's constructs will help to identify vulnerable features of the implementation process with respect to the work involved in introducing and embedding the RETAKE intervention and the importance and influence of contextual factors on trial outcomes.

Investigating the implementation fidelity of a complex intervention offers insight into barriers and facilitators to delivery to inform future study design. It also yields valuable information regarding the 'core components' and 'active ingredients' of an intervention and any permitted modifications for clinical implementation.[37]

For peer review only - http://bmjopen.bmj.com/site/about/guidelines.xhtml

Understanding of the causal mechanisms of complex interventions is vital in being able to deliver an effective intervention in other settings. This process evaluation will measure these modifications and their effect on the intervention's fidelity while providing the context in which to interpret the variation in outcomes on the effectiveness of the trial.

Ethics and dissemination

Ethics approval has been obtained through the East Midlands – Nottingham 2 Research Ethics Committee (REC) (Ref: 18/EM/0019) and the NHS Health Research Authority.

Availability of data and materials

No additional data will be made available.

Competing interests

The authors declare that they have no competing interests.

Funding

This study is funded by the NIHR HTA programme (ref: 15/130/11). The views expressed herein are those of the authors, not necessarily the NIHR, the Department of Health and Social Care, or the NHS.

Authors' contributions

KR, CM, AFa, AB, ROC, MW, and CW conceived the study. KR, DJC, and CM designed the process evaluation. KR, CM, DJC, SC, KC, JH, JP and KP

For peer review only - http://bmjopen.bmj.com/site/about/guidelines.xhtml

operationalized the process evaluation protocol. KR, JP, and JH designed the intervention. AS has the role of trial sponsor. IH, RB, and AFa devised the data management and statistical analysis plan. JS and JM acted as PPI collaborators to support plans for trial design/delivery, management, and dissemination of trial findings. VM and SH have responsibility for management of the trial. KR, SC and DJC drafted the manuscript; all other authors read and approved the final version.

Authors' information

[1]Associate Professor of Rehabilitation Research, Division of Rehabilitation, Ageing and Wellbeing, School of Medicine, B-Floor, Medical School Queen's Medical Centre, Nottingham NG7 2UH.

[2]Clinical Trials Research Unit (CTRU), Leeds Institute of Clinical Trials Research, Level 11 Worsley Building, University of Leeds, Leeds LS2 9JT.

[3]Health Economics Group, Room 2.37, Norwich Medical School, University of East Anglia, Norwich Research Park, Norwich NR4 7TJ. [4]Lancashire Clinical Trials Unit, Lancashire Applied health Research Collaboration Hub, Brook Building, Room 429, University of Central Lancashire, Preston PR1 2HE. [5] Academic Department of Rehabilitation Medicine, Leeds Institute of Molecular Medicine, University of Leeds, Level D, Martin Wing, Leeds General Infirmary, Leeds LS1 3EX. [6]Patient and Public Involvement Collaborator, Hampshire, UK. [7]Different Strokes, Raphael House, Ilford, London IG1 1YT. [8]Geoffrey Jefferson Brain Research Centre, The Manchester Academic Health Science Centre, Northern Care Alliance & University of Manchester, Manchester M13 9PL. [9]Department of Population Health Sciences, Faculty of Life Sciences & Medicine, King's College London, 5th Floor Addison

For peer review only - http://bmjopen.bmj.com/site/about/guidelines.xhtml

House, Guy's Campus, London SE1 1UL. [10]Research and Innovation, Jubilee Conference Centre, Jubilee Campus, Wollaton Road, Nottingham NG8 1BB.

[11]Visiting Associate Professor in Stroke Care Academic Unit for Ageing and Stroke Research, Leeds Institute of Health Sciences

University of Leeds, Leeds LS2 9JT,UK.

For peer review only

REFERENCES

1. Stroke Association. State of the Nation: stroke statistics. Stroke Association. 2018.https://www.stroke.org.uk/resources/state-nation-stroke-statistics (accessed 27 Apr 2021).
2. Daniel K, Wolfe CD, Busch MA, et al. What are the social consequences of stroke for working-aged adults? A systematic review. *Stroke*2009;40:e431-40.

For peer review only - http://bmjopen.bmj.com/site/about/guidelines.xhtml

3. Sinclair E, Radford K, Grant M, et al. Developing stroke-specific vocational rehabilitation: a soft systems analysis of current service provision. *Disabil Rehabil*2014;36:409-17.
4. Corr S, Wilmer S. Returning to work after a stroke: an important but neglected area. *Br J Occup Ther*2003;66:186-92.
5. Frank AO, Thurgood J. Vocational rehabilitation in the UK: opportunities for health-care professionals. *Int J Ther Rehabil*2006;13:126-34.
6. Lindström B, Röding J, Sundelin G. Positive attitudes and preserved high level of motor performance are important factors for return to work in younger persons after stroke: a national survey. *J Rehabil Med*2009;41:714-8.
7. Palstam A, Westerlind E, Persson HC, et al. (2019). Work-related predictors for return to work after stroke. *Acta Neurol Scand*2019;139:382-388. doi:10.1111/ane.13067
8. Waddell G, Burton AK, Kendall NA. Vocational rehabilitation–what works, for whom, and when? (Report for the Vocational Rehabilitation Task Group). London: TSO 2008.
9. Escorpizo R, Reneman MF, Ekholm J, et al. A conceptual definition of vocational rehabilitation based on the ICF: building a shared global model. *J Occup Rehabil* 2011;21:126-33.
10. Ntsiea MV, Van Aswegen H, Lord S, et al. The effect of a workplace intervention programme on return to work after stroke: a randomised controlled trial. *Clin Rehabil*2015;29:663-73.
11. Trexler LE, Trexler LC, Malec JF, et al. Prospective randomized controlled trial of resource facilitation on community participation and vocational outcome following brain injury. *J Head Trauma Rehabil*2010;25:440-6.

For peer review only - http://bmjopen.bmj.com/site/about/guidelines.xhtml

12. Radford K, King K, McLellan V, et al. An individually randomised controlled multi-centre pragmatic trial with embedded economic and process evaluations of early vocational rehabilitation compared with usual care for stroke survivors: study protocol for the RETurn to work After stroKE (RETAKE) trial. Submitted to Trials 2020.

13. Grant M. *Developing, delivering and evaluating stroke specific vocational rehabilitation: A feasibility randomised controlled trial (Doctoral dissertation, University of Nottingham)*. 2016.

14. Lynch EA, Chesworth BM, Connell LA. Implementation—The missing link in the research translation pipeline: is it any wonder no one ever implements evidence-based practice?. *Neurorehabil Neural Repair*2018;32:751-61.

15. Glasziou P, Straus S, Brownlee S, et al. Evidence for underuse of effective medical services around the world. *Lancet*2017;390:169-177. doi:10.1016/S0140-6736(16)30946-1

16. Walker MF, Hoffmann TC, Brady MC, et al. Improving the development, monitoring and reporting of stroke rehabilitation research: Consensus-based core recommendations from the Stroke Recovery and Rehabilitation Roundtable. *Int J Stroke*2017;12:472-9.

17. Moore GF, Audrey S, Barker M, et al. Process evaluation of complex interventions: Medical Research Council guidance. *BMJ*2015;350:h1258.

18. Basch CE, Sliepcevich EM, Gold RS, et al. Avoiding type III errors in health education program evaluations: a case study. *Health Educ Q*1985;12:315-31.

19. Dusenbury L, Brannigan R, Falco M, et al. A review of research on fidelity of implementation: implications for drug abuse prevention in school settings. *Health Educ Res*2003;18:237-56.

For peer review only - http://bmjopen.bmj.com/site/about/guidelines.xhtml

20. Colditz GA, Emmons KM. The promise and challenges of dissemination and implementation research. *Dissemination and implementation research in health: Translating science to practice*2012;24:3-22.

21. Bosch M, VanDerWeijden T, Wensing M, et al. Tailoring quality improvement interventions to identified barriers: a multiple case analysis. *J Eval Clin Pract*2007;13:161-8.

22. Levac D, Glegg SM, Sveistrup H, et al. A knowledge translation intervention to enhance clinical application of a virtual reality system in stroke rehabilitation. *BMC Health Serv Re*s2016;16:557.

23. Ioannidis JP, Greenland S, Hlatky MA, et al. Increasing value and reducing waste in research design, conduct, and analysis. *Lancet*2014;383:166-75.

24. May C, Murray E, Finch T, et al. Normalization process theory online users' manual and toolkit. NPT: Normalization Process Theory. 2010. http://www.normalizationprocess.org/ (accessed 27 Apr 2021).

25. Carroll C, Patterson, M, Wood, S, et al. A conceptual framework for implementation fidelity. *Implementation Sci*2007;2:40.

26. Ciesielska M, Boström KW, Öhlander M. Observational methods. In: Ciesielska M, ed. Qualitative Methodologies in Organization Studies Volume II: Methods and Possibilities. Cham, Switzerland. Springer 2018:33-52.

27. Braun V, Clarke V. Using thematic analysis in psychology. *Qual Res Psychol* 2006;3:77-101.

28. Hoffmann TC, Glasziou PP, Boutron I, et al. Better reporting of interventions: template for intervention description and replication (TIDieR) checklist and guide. *BMJ* 2014;348:g1687.

For peer review only - http://bmjopen.bmj.com/site/about/guidelines.xhtml

29. Masterson-Algar P, Burton CR, Brady MC, et al. The PD COMM trial: a protocol for the process evaluation of a randomised trial assessing the effectiveness of two types of SLT for people with Parkinson's disease. *Trials*2017;18:397.

30. Clarke DJ, Godfrey M, Hawkins R, et al. Implementing a training intervention to support caregivers after stroke: a process evaluation examining the initiation and embedding of programme change. *Implementation Sci*2013;8:96 doi:10.1186/1748-5908-8-96

31. Clarke DJ, Hawkins R, Sadler E, et al. Introducing structured caregiver training in stroke care: findings from the TRACS process evaluation study. *BMJ Open*2014;4:e004473 doi:10.1136/bmjopen-2013-004473

32. Luker JA, Craig LE, Bennett L, et al. Implementing a complex rehabilitation intervention in a stroke trial: a qualitative process evaluation of AVERT. *BMC Med Res Methodol* 2016; 52 doi:10.1186/s12874-016-0156-9

33. Liu H, Lindley R, Alim M, et al. Protocol for process evaluation of a randomised controlled trial of family-led rehabilitation post stroke (ATTEND) in India. *BMJ Open*2016;6:e012027 doi:10.1136/ bmjopen-2016-012027

34. Liu H, Lindley R, Alim M, et al. Family-led rehabilitation in India (ATTEND)—Findings from the process evaluation of a randomized controlled trial. *Int J Stroke*2019;53–60 doi:10.1177/1747493018790076

35. Salbach NM, Wood-Dauphinee S, Desrosiers J, et al. Facilitated interprofessional implementation of a physical rehabilitation guideline for stroke in inpatient settings: process evaluation of a cluster randomized trial. *Implementation Sci* 2017;100 doi:10.1186/s13012-017-0631-7

For peer review only - http://bmjopen.bmj.com/site/about/guidelines.xhtml

36. Radford K, Sutton C, Sach T, et al. FRESH – Facilitating Return to work through Early Specialist Health-based interventions: feasibility randomised controlled trial. *Health Technology Assessment*2018;22 doi:10.3310/hta22330

37. Hart T, Whyte J, Dijkers M, et al. Manual of Rehabilitation Treatment Specification. http://mrri.org/innovations/manual-for-rehabilitation-treatment-specification (accessed 27 Apr 2021).

For peer review only

For peer review only - http://bmjopen.bmj.com/site/about/guidelines.xhtml

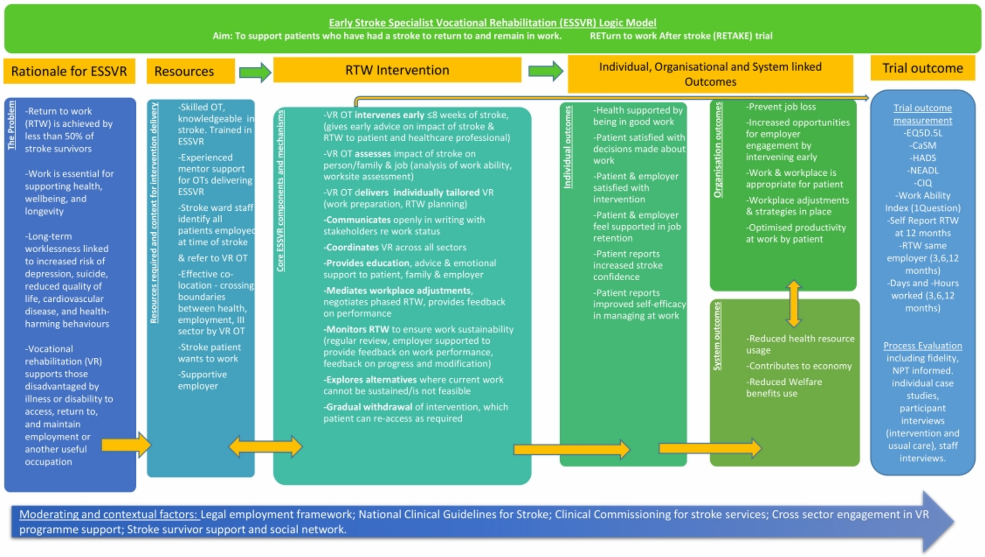

Figure 1. The ESSVR logic model.

338x190mm (300 x 300 DPI)

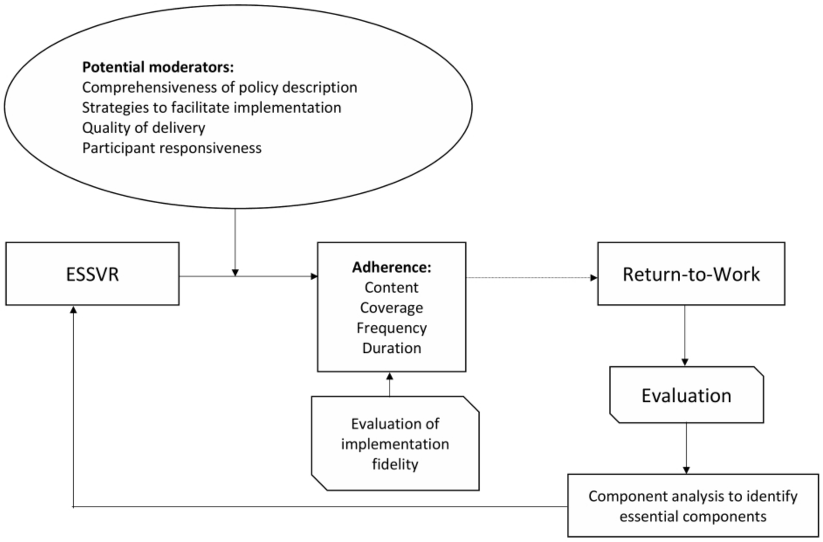

Figure 2. Assessment of fidelity and factors moderating ESSVR delivery in accordance with the Conceptual Framework for Implementation Fidelity.[25]

297x210mm (300 x 300 DPI)

BMJ Open

The RETurn to work After stroKE (RETAKE) Trial: protocol for a mixed-methods process evaluation using normalisation process theory

Journal:	*BMJ Open*
Manuscript ID	bmjopen-2021-053111.R1
Article Type:	Protocol
Date Submitted by the Author:	23-Jan-2022
Complete List of Authors:	Radford, Kathryn A; University of Nottingham Faculty of Medicine and Health Sciences, Centre for Rehabilitation and Ageing Research<br>McKevitt, Christopher; King's College London, Department of Public Health Sciences<br>Clarke, Sara; University of Nottingham, Division of Rehabilitation, Aging and Wellbeing<br>Powers, Katie; University of Nottingham<br>Phillips, Julie; University of Nottingham<br>Craven, Kristelle; University of Nottingham<br>Watkins, Caroline; University of Central Lancashire, Faculty of Health and Wellbeing<br>Farrin, Amanda; University of Leeds, Clinical Trials Research Unit<br>Holmes, Jain; University of Nottingham<br>Cripps, Rachel; King's College London<br>McLellan, Vicki; University of Leeds, Clinical Trials Unit<br>Sach, Tracey; University of East Anglia, School of Chemical Sciences and Pharmacy<br>Brindle, Richard; University of Leeds, Clinical Trials Unit<br>Holloway, Ivana; University of Leeds, Clinical Trials Unit<br>Hartley, Suzanne; University of Leeds, Clinical Trials Research Unit<br>Bowen, Audrey; The University of Manchester, DNEP, SBS, FBMH<br>O'Connor, Rory J.; University of Leeds<br>Stevens, Judith; University of Nottingham<br>Walker, Marion; University of Nottingham<br>Murray, John; University of Nottingham<br>Shone, Angela; University of Nottingham<br>Clarke, David; University of Leeds, Academic Unit of Elderly Care and Rehabilitation
**Primary Subject Heading**:	Cardiovascular medicine
Secondary Subject Heading:	Rehabilitation medicine, Research methods, Qualitative research, Health services research, Occupational and environmental medicine
Keywords:	Stroke < NEUROLOGY, Clinical trials < THERAPEUTICS, STROKE MEDICINE

TITLE

The RETurn to work After stroKE (RETAKE) Trial: protocol for a mixed-methods process evaluation using normalisation process theory

NAMES OF PROTOCOL CONTRIBUTORS

Kathryn A Radford[1*], Christopher McKevitt[9], Sara Clarke[1], Katie Powers[1], Julie Phillips[1], Kristelle Craven[1], Caroline Watkins[4], Amanda Farrin[2] , Jain Holmes[1], Rachel Cripps[9], Vicki McLellan[2], Tracey H Sach[3], Richard Brindle[2], Ivana Holloway[2], Suzanne Hartley[2], Audrey Bowen[8], Rory O'Connor[5], Judith Stevens[6], Marion Walker[1], John Murray[7], Angela Shone[10], David J Clarke[11]

*Correspondence: Kathryn.radford@nottingham.ac.uk

[1]Centre for Rehabilitation and Ageing Research, School of Medicine, B-Floor, Medical School Queen's Medical Centre, Nottingham NG7 2UH. Full details of authors' information are presented at the end of the article.

For peer review only

Authors' information

[1]Professor of Rehabilitation Research, Centre for Rehabilitation and Ageing Research, School of Medicine, B-Floor, Medical School Queen's Medical Centre, Nottingham NG7 2UH.

[2]Clinical Trials Research Unit (CTRU), Leeds Institute of Clinical Trials Research, Level 11 Worsley Building, University of Leeds, Leeds LS2 9JT.

[3]Health Economics Group, Room 2.37, Norwich Medical School, University of East Anglia, Norwich Research Park, Norwich NR4 7TJ. [4]Lancashire Clinical Trials Unit,

For peer review only - http://bmjopen.bmj.com/site/about/guidelines.xhtml

Lancashire Applied health Research Collaboration Hub, Brook Building, Room 429, University of Central Lancashire, Preston PR1 2HE. [5] Academic Department of Rehabilitation Medicine, Leeds Institute of Molecular Medicine, University of Leeds, Level D, Martin Wing, Leeds General Infirmary, Leeds LS1 3EX. [6]Patient and Public Involvement Collaborator, Hampshire, UK. [7]Different Strokes, Raphael House, Ilford, London IG1 1YT. [8]Geoffrey Jefferson Brain Research Centre, The Manchester Academic Health Science Centre, Northern Care Alliance & University of Manchester, Manchester M13 9PL. [9]Department of Population Health Sciences, Faculty of Life Sciences & Medicine, King's College London, 5th Floor Addison House, Guy's Campus, London SE1 1UL. [10]Research and Innovation, Jubilee Conference Centre, Jubilee Campus, Wollaton Road, Nottingham NG8 1BB.

[11]Visiting Associate Professor in Stroke Care Academic Unit for Ageing and Stroke Research, Leeds Institute of Health Sciences
University of Leeds, Leeds LS2 9JT, UK.

WORD COUNT: 3985 (minus abstract, strengths and limitations, tables/figures and references)

For peer review only - http://bmjopen.bmj.com/site/about/guidelines.xhtml

ABSTRACT

Objectives

This mixed-method process evaluation underpinned by Normalisation Process Theory (NPT) aims to measure fidelity to the intervention, understand the social and structural context in which the intervention is delivered, and identify barriers and facilitators to intervention implementation.

Setting

Return to Work after Stroke (RETAKE) is a multi-centre individual patient randomised controlled trial to determine whether Early Stroke Specialist Vocational Rehabilitation (ESSVR) plus usual care is a clinically and cost-effective therapy to facilitate return to work after stroke, compared with usual care alone. This protocol paper describes the embedded process evaluation.

Participants and outcome measures

Intervention training for therapists will be observed and use of remote mentor support reviewed through documentary analysis. Fidelity will be assessed through participant questionnaires and analysis of therapy records, examining frequency, duration and content of ESSVR sessions. To understand the influence of social and structural contexts, the process evaluation will explore therapists' attitudes towards evidence-based practice, competency to deliver the intervention and evaluate potential sources of contamination. Longitudinal case studies incorporating non-participant observations will be conducted with a proportion of intervention and usual care participants. Semi-structured interviews with stroke survivors, carers, occupational therapists, mentors, service managers and employers will explore their

For peer review only - http://bmjopen.bmj.com/site/about/guidelines.xhtml

experiences as RETAKE participants. Analysis of qualitative data will draw on thematic and Framework approaches. Quantitative data analysis will include regression models and descriptive statistics. Qualitative and quantitative data will be independently analysed by process evaluation and Clinical Trials Research Unit teams respectively. Linked data, e.g. fidelity and describing usual care will be synthesised by comparing and integrating quantitative descriptive data with the qualitative findings.

Ethics and dissemination

Approval obtained through the East Midlands – Nottingham 2 Research Ethics Committee (Ref: 18/EM/0019) and the National Health Service (NHS) Research Authority. Dissemination via journal publications, stroke conferences, social media and meetings with national Stroke clinical leads.

Trial registration

Registration number: ISRCTN: 12464275

KEYWORDS

Return to work, stroke, vocational rehabilitation, occupational therapy, complex intervention, process evaluation, randomised controlled trial, mixed-methods, qualitative, Normalisation Process Theory, Consolidated Framework for Implementation Fidelity

For peer review only - http://bmjopen.bmj.com/site/about/guidelines.xhtml

For peer review only

STRENGTHS AND LIMITATIONS OF THIS STUDY

- A mixed-methods theory-driven process evaluation will generate detailed findings to assist in interpreting the results of a pragmatic, multi-centre individual patient randomised controlled trial of a complex vocational rehabilitation intervention, which crosses the work/health divide.
- This is one of the most comprehensive multi-site, multi-component, multi-stakeholder perspective process evaluations embedded in a stroke rehabilitation trial, involving detailed assessment of implementation fidelity, therapist competency to deliver the trial intervention, contamination logging and exploration of social and structural influences on intervention provision in post-stroke rehabilitation services.
- Longitudinal case studies with intervention and usual care will capture participant experiences of providing and experiencing the intervention including those of employers.
- The Covid19 pandemic limited researcher access to direct observation of face-to-face intervention delivery and employer interactions with stroke survivors in each site. Integration of interview data from different participant sources including stroke survivors and carers, occupational therapists and employers with available observational data is planned to address this limitation.

For peer review only - http://bmjopen.bmj.com/site/about/guidelines.xhtml

BACKGROUND

Approximately 100,000 people in the UK suffer from a stroke every year,[1] and around 1 in 4 are of working age.[2] Returning to work after a stroke is a major goal for stroke survivors, contributing to social identity, emotional and financial wellbeing, and conferring a sense of purpose and has benefits for the individual, the individual's family and the economy.[3] Despite this, only half of working age stroke survivors make a successful return to meaningful work, and they are two to three times more likely to be unemployed eight years after their stroke than the general population.[1] Although impairments in the stroke survivor's physical, cognitive and communication abilities can affect this,[4-5] social and environmental factors such as personal and employer beliefs and attitudes, job type and organisation size and the benefits system also play an important part.[6-7]

Vocational rehabilitation (VR) is defined as whatever helps someone with a health problem to return to, or remain in, work and includes both work *and* work-related education.[8] It involves helping people find work, helping those who are in work but having difficulty, as well as supporting career progression in spite of illness or disability. The primary aim is to optimise work participation.[9] Existing research suggests that VR may help stroke survivors return to their previous job or find new work,[10-11] however trials to date involve small samples in non-UK settings.

RETAKE is a multi-centre individual patient randomised controlled trial (RCT) which aims to determine the clinical and cost-effectiveness of an Early Stroke Specialist Vocational Rehabilitation (ESSVR) intervention in addition to usual NHS rehabilitation on stroke survivors' return to work at 12 months post-randomisation, compared to NHS

For peer review only - http://bmjopen.bmj.com/site/about/guidelines.xhtml

rehabilitation alone.[12] Acceptability and utility were assessed in a feasibility trial.[13] ESSVR combines conventional occupational therapy (OT) with case coordination. The intervention commences within two weeks of randomization and lasts up to 12 months post-randomization. It is intended for delivery in the community as often as required by individuals, as determined by a stroke specialist OT with additional VR training. ESSVR includes the following: (a) assessing stroke impact on the person and their job; (b) educating individuals, employers, and families about stroke impact on work, and strategies to lessen impact (e.g., memory aids, fatigue management); (c) work preparation, including opportunities to practice work skills; and (d) liaison with employers to plan and monitor a phased return to work (RTW) (see Appendix I). The target number of participants for the trial is 760 participants (420 ESSVR and 340 usual care) from 20 UK hospitals and linked early supported discharge/community services. The RETAKE trial and embedded process evaluation commenced in June 2018 and will complete in March 2022. This period includes a funder approved extension of seven months necessitated by an unplanned pause in recruitment during the Covid19 pandemic.

Failure to implement evidence-based stroke rehabilitation interventions in clinical practice may result in unnecessary suffering and disability.[14-15] Trialists must consider future implementation in the real world when designing clinical trials, paying particular attention to the context for intervention delivery and factors likely to influence its uptake and use.[16] This is especially true for trials of complex rehabilitation interventions, which comprise multiple interacting components, and target a number of different organisational levels, making them particularly challenging to implement. An embedded process evaluation provides for an in-depth exploration of factors influencing the implementation of complex interventions.

For peer review only - http://bmjopen.bmj.com/site/about/guidelines.xhtml

The Medical Research Council (MRC) argue for a systematic approach to designing and conducting process evaluations, drawing on clear descriptions of intervention theory and the identification of key process questions.[17] Mixed methods approaches to process evaluation are increasingly common and consistent with the MRC framework's emphasis on exploring and understanding the important relationship between context, mechanisms and implementation. Theory driven process evaluations are recommended alongside complex intervention trials to measure what is delivered. These measurements include fidelity (whether the intervention was delivered as intended), dose (the quantity of intervention implemented), and "reach" of interventions to understand how the intended audience interacts with the intervention.[17] Fidelity data are necessary to interpret intervention outcomes, but despite an extensive literature supporting its importance, fidelity is commonly under-reported in studies of complex rehabilitation interventions. Whilst most trials of VR have not raised particular concerns about fidelity, ESSVR in the RETAKE trial is an example of a particularly complex intervention that crosses organisational boundaries, involves interactions between multiple stakeholders, is highly individually tailored and requires behavioural change by the patient, their family and employer. Therefore, in the process evaluation for the RETAKE trial we have included specific methods to measure fidelity. Alongside a focus on fidelity, in-depth qualitative exploration of participants' experiences of an intervention, and of the social and structural context in which an intervention is provided, are essential elements of process evaluation of complex interventions. This ensures any adaptations made to tailor intervention to the individual and/or differing contexts, which might undermine fidelity can be evaluated. Understanding and reporting how

For peer review only - http://bmjopen.bmj.com/site/about/guidelines.xhtml

the intervention (including training and support, communication and management structures) is delivered is important for replication in clinical practice.[17] Such evaluation aims to reduce the chance of discounting effective interventions (Type II error) or erroneously attributing outcomes to treatment effectiveness, when interventions are not delivered as intended (Type III Errors).[18 - 21] The approach is designed to improve trial design and knowledge translation interventions enhancing clinical implementation and reducing research waste.[22-23]

This paper reports the protocol for the process evaluation embedded in the RETAKE trial.

AIMS AND OBJECTIVES

Aims

To measure fidelity to the ESSVR intervention and understand the social and structural context in which the intervention is delivered and identify factors which may influence the quality of implementation.

Objectives

Fidelity measurement and competency assessment will

1. Ascertain intervention dose
2. Describe content of usual care and ESSVR
3. Describe levels of adherence to the ESSVR intervention

For peer review only - http://bmjopen.bmj.com/site/about/guidelines.xhtml

4. Understand the delivery of Usual Care and ESSVR.

5. Determine OTs competency to deliver ESSVR

Social and structural context will include

6. Describe participating sites.

7. Understand professionals' experiences of being trained to deliver the intervention.

8. Understand experiences of delivering the intervention.

9. Understand the social and structural factors which support or act as barriers to the implementation of the intervention.

10. Understand participants' experience of being supported to return to work after stroke.

11. Identify potential contaminants

METHODS

Design

Embedded theory-driven mixed-methods process evaluation incorporating qualitative and quantitative methods. The process evaluation will draw on the intervention logic model developed by the Trialists (Figure 1) and will be underpinned by Normalisation Process Theory (NPT), an implementation theory built on four constructs (coherence, cognitive participation, collective action and reflexive monitoring) each informed by four components.[24] NPT will be used in the development of data collection tools (interview topic guides and observation checklists [see Table 1]) and as a sensitising lens in qualitative data analysis and interpretation. NPT constructs

For peer review only - http://bmjopen.bmj.com/site/about/guidelines.xhtml

236 will underpin the process evaluation and provide insights into the implementation and
237 integration of the intervention into participating stroke services. This will include how
238 the intervention is received, understood, implemented and how it could be
239 normalised into the current healthcare system.

240 **Table 1: Examples of question topics related to NPT constructs**

**Normalisation Process Theory Constructs and components**	**NHS Staff/ therapist interview topics** (some may also arise in informal feedback during training observations)	**Stroke Participant interview topics** (some may also arise in intervention / usual care observations)	**Employer interview topics**
**Coherence:**<br>• Differentiation<br>• Communal specification<br>• Individual specification<br>• Internalisation	How do staff describe the intervention?<br><br>How is the intervention similar to/different from usual care?<br><br>Who would (most) benefit from the intervention?	Experiences of RTW support received: similarities/differences between control and intervention participants	Experience of liaising with the therapist and/or participant on RTW issues
**Cognitive participation**<br>• Initiation<br>• Enrolment<br>• Legitimation<br>• Activation	Do staff see value/potential in the intervention?<br><br>Have they found the training and experience a worthwhile investment of time?<br><br>Do they feel they have the competence/ resources to deliver the intervention effectively?	What were their expectations? Did patients (& carers) value the intervention?<br><br>How did they respond to the therapists' suggestions?<br><br>Did they feel they had the ability/resources/confidence to progress through the sessions and ultimately RTW?<br><br>Context in which participant received RETAKE/acted on suggestions: social, financial, health state, access to opportunities	Expectations of the processes: liaising with therapist/patient and patient's RTW<br><br>(Prior) experience in supporting RTW for people with disabilities
**Collective action**<br>• Interactional workability<br>• Relational integration	How compatible is the intervention with the existing stroke care pathway?<br><br>What other RTW	How did participants accommodate the intervention sessions/follow up actions?<br><br>How did they manage/are	Views on who is responsible /roles in supporting RTW<br><br>Financial implications e.g. modifications

For peer review only - http://bmjopen.bmj.com/site/about/guidelines.xhtml

• Skill set workability<br>• Contextual integration	services/resources exist locally? How does this intervention compare/complement those services? Describe working relationships with those services.<br><br>Support from managers and colleagues during the intervention period	they managing their RTW (if applicable)?<br><br>Financial implications	
**Reflexive monitoring**<br><br>• Systematisation<br>• Communal appraisal<br>• Individual appraisal<br>• Reconfiguration<br>•	Perceived effects on patients (& carers)<br><br>Views on time/resources invested in delivery vs impact<br><br>What is needed to make it possible to roll out the intervention effectively? (Changes to intervention; changes in services/resources needed for delivery)	Perceived effects of RETAKE/other RTW support<br><br>Views on time/resources invested in participation vs impact<br><br>What was good about RETAKE and what could be improved? (Content of intervention sessions/work plans, timing, relationship with therapist)	Perceptions of benefit to employer/tutor/advisor<br><br>Perceptions of benefit to employee<br><br>What was helpful about discussions with therapist/participant?<br><br>What further information/support would they have liked – at what time?

Figure 1. The ESSVR logic model.

Column 3 of the logic model identifies the core components of the ESSVR intervention. A more detailed description of the development and feasibility testing of the ESSVR intervention has been published previously. [13]

In addition, the Conceptual Framework for Implementation Fidelity (CFIF) (Figure 2) will guide collection and analysis of quantitative data.[25] The CFIF outlines the components and variables that make up and affect intervention fidelity and explains

For peer review only

For peer review only - http://bmjopen.bmj.com/site/about/guidelines.xhtml

how they relate to each other. Adherence includes content and dose (frequency, coverage and duration) of the delivery.[25]

Figure 2. Assessment of fidelity and factors moderating ESSVR delivery in accordance with the Conceptual Framework for Implementation Fidelity.[25]

Eligibility criteria

Stroke survivors that meet the following criteria for inclusion in the RETAKE trial will be eligible to participate in the process evaluation:

- Age ≥18 years.
- Admitted to hospital with new stroke (all severities).
- In work at stroke onset (including self-employed, paid or voluntary).
- Willing and have capacity to provide informed consent to participate in the study.
- Have sufficient proficiency in English to contribute to the data collection required for research.

Potential participants who do not intend to return to work will be excluded. Potential participants with a transient ischaemic attack will be excluded.

Inclusion criteria for carers of potential participants:

- Nominated carer of consenting participant.
- Willing and have capacity to provide informed consent to participate in the study.
- Have sufficient proficiency in English to contribute to the data collection required for research.

For peer review only - http://bmjopen.bmj.com/site/about/guidelines.xhtml

Informed Consent

Potential participants will be provided with an information sheet and be provided the opportunity to ask questions of a researcher prior to consent. Written informed consent will be obtained from all participants. When a participant is randomised to the case study element, a researcher will contact the participant to gain consent for interview and observations. Consent will be reaffirmed at the start of interviews. This process will be the same for carer, employer, OT and NHS staff interviews. For employer interviews, additional consent to contact the employer will be requested from the case study participant before the employer is contacted. OTs who will deliver the ESSVR intervention and mentors supporting these OTs will be recruited prior to intervention training. NHS staff involved in the management, commissioning or delivery of stroke rehabilitation in each site participating in the RETAKE trial will be recruited.

Sampling

For professional and patient interviews, as far as possible we will use a purposive sampling strategy to ensure diversity in terms of geographical location (e.g. urban vs rural centres), level of staff seniority and participant sociodemographic variables (including gender and socio-economic status). See Table 2 for the timepoints at which data collection is planned.

Patient and Public Involvement Statement

Stroke survivors are involved in all stages of the research cycle.

Design and development.

For peer review only - http://bmjopen.bmj.com/site/about/guidelines.xhtml

Two stroke survivors are co-applicants on the grant and assisted in identifying the research questions, designing the study and developing the trial protocol.

Delivery.

Two PPI are members of the Trial Steering Committee, and two are members of the Trial Management Group. Additionally, our RETAKE PPI (Patient & Public Involvement) group, which has six members, meets quarterly. Examples of the work achieved by the PPI group to date are:

- Helping define the primary outcome and defining ‘voluntary work’ which is included in the definition of the primary outcome.
- Evaluating all patient facing material including aphasia friendly recruitment material.
- Co-development of interview topic guides for trial participants and occupational therapists.
- Overcoming problems with recruitment. For example, resources and narratives to assist recruiters in approaching people with severe stroke.
- Assisting in the design of new materials to promote follow up e.g. including a ‘patient journey leaflet’ and Thankyou cards.
- Helping reduce the length of follow-up questionnaires.
- Advising on communicating with participants during the pandemic.
- Changes to the Excess Treatment Cost payment models during trial, caused problems for the study. One PPI member wrote directly to Directors of the NIHR, NHS England, Health and Social Care and the leads for the NIHR Clinical Research Network to explain the impact that these changes on the trial. She received a prompt response which was extremely helpful to the research team. This has assisted us in explaining the new system to clinical colleagues and researchers in the Trusts.
- Co-Development of a trial website and trial newsletters.

A draft report on the process evaluation findings will be presented to the PPI group for their consideration and comments prior to submission of the final report to the funder and as part of planning publications and dissemination. The PPI group will be involved in writing up and presenting study findings.

For peer review only - http://bmjopen.bmj.com/site/about/guidelines.xhtml

Data Collection

The process evaluation will employ qualitative and quantitative methods to address the research questions. Table 2 illustrates the relationship between the process evaluation aims, research questions, data sources and data collection methods. The following section describes each data source in more detail.

Table 2: RETAKE process evaluation research questions and data sources

Aims	Research questions	Data Source(s)	Method(s)	Timepoint
**Measure fidelity to the intervention**	What is the intervention dose, intensity and duration?	• Intervention content case report forms (CRFs)	Quantitative	Months 3-45
	What is the (reported) content of the ESSVR intervention?<br><br>What is the content of usual care?	• Intervention content CRFs.<br>• NHS therapy records.<br>• Stroke survivor-reported resource use data.<br>• Stroke survivor carer and OT interviews	Quantitative and qualitative	Months 3-45<br><br>Months 12-45<br><br>Months 12-36
	Was the intervention delivered with fidelity?<br><br>What factors affect implementation fidelity?<br><br>Are RETAKE OTs competent to deliver the ESSVR intervention?	• Fidelity checklist,<br>• Intervention content CRFs<br>• Mentoring records,<br>• RETAKE OT interviews<br><br>• Individual OT performance in assessed vignettes at	Quantitative and qualitative<br><br>Quantitative	Months 3-45<br><br>Months 12-18<br><br>Months 1-8 and as new OT join the trial and 6 and 12 months post training.

For peer review only - http://bmjopen.bmj.com/site/about/guidelines.xhtml

		baseline and 6 months<br>• RETAKE OT case record reviews at 12 months post training		
**Understand the social and structural context which may influence intervention implementation and future embedding in practice settings.**	What is the context for intervention delivery?	• Site survey at baseline, mid-point and end of intervention delivery	Quantitative and qualitative	Months 1, 18 and 36*<br>*later timepoint for end of intervention delivery where sites recruit beyond the Covid19 extension.
	What services are in place for supporting patients in return to work?	• Site survey at baseline, mid-point and end of intervention delivery	Quantitative and qualitative	As above.
	What are the staffing levels at sites?	• Site survey at baseline, mid-point and end of intervention delivery	Quantitative and qualitative	As above
	Potential for contamination: Are there proposed or actual VR service developments or changes in practice in place/ planned at site?	• Site survey at baseline, mid-point and end of intervention delivery<br>• NHS staff interviews	Quantitative and qualitative	As above.
	What are the RETAKE OTs' perceptions of training and mentoring to deliver the intervention?	• Observations at training sessions<br>• RETAKE OT interviews	Qualitative	Months 1-8 and as new OT join the trial.
	How do OTs experience delivering the intervention?	• Observations of ESSVR sessions<br>• RETAKE OT interviews<br>• Mentoring records	Qualitative	Months 12-18<br><br>Months 12-18<br>Months 12-45
	What are the social and structural factors supporting or acting as barriers to intervention implementation?	• Observations of usual care and ESSVR sessions<br>• RETAKE OT interviews	Qualitative	Months 1-8<br><br>Months 12-18<br>Months 12-18<br><br>Months 12-24

For peer review only

		• Usual Care therapist interviews<br>• NHS Staff interviews<br>• Mentor interviews		Months 6-8
	How do participants' experience being supported to return to work after stroke?	• Stroke survivor interviews<br>• Carer interviews<br>• Employer interviews	Qualitative	Months 12-24<br><br>Months 12-24<br>Months 12-24

Intervention content Case Report Forms (CRFs)

To check on fidelity in terms of (early) intervention within two weeks of recruitment, initial Session CRFs (one per participant) record the Intervention start date and whether this occurred within 8 weeks of stroke. Participant Summary CRFs record the number of sessions attended out of those proposed and whether there was an agreed ending for the OT led return to work support. To ascertain intervention dose and describe intervention content, data will be extracted from intervention CRFs for all participants (see Table 3). Therapists record each intervention session against pre-defined components, on an 'Intervention content CRF'.[13] These data will be used to identify which components of the intervention were delivered, to what extent therapists adhered to the intervention process described in the RETAKE manual, and to what extent participants adhered to the intervention. For case study participants only, content data will be cross-referenced with the OT's clinical case notes and additional data extracted to explain how the RETAKE intervention interacts with usual care and other services such as employment services. Participants' consent includes permission for members of the trials team to access their therapy records.

For peer review only - http://bmjopen.bmj.com/site/about/guidelines.xhtml

Describing usual care

To describe the content of the intervention and of usual care, resource use questions pertaining to participants' use of health and social care services over the previous three months will be completed by all participants at three, six- and twelve-months post-randomisation as part of follow-up. This data will be used to describe the content of usual care, and in case study participants (n=38) will be triangulated with therapists' clinical notes and participant interview transcripts.

For peer review only - http://bmjopen.bmj.com/site/about/guidelines.xhtml

Fidelity

To assess implementation fidelity a range of data collection methods informed by the CFIF will be used (see Table 3).[25]

Therapist competency assessment

Following attendance at a two-day, manualised face-to-face training session with VR expert trainers and again at refresher training six months later, retake OTs competence will be assessed using OTs written responses to questions based on vignettes depicting novel RTW after stroke scenarios. Model answers developed by the training team will be used to measure competence using criteria based on knowledge of the intervention process (40%), clinical reasoning (50%) and written communication (10%). Scores will be mapped to a rubric identifying OTs as highly competent (≥70%), competent (50-69%) or needing additional support (≤49%) (see Appendix II). In addition, as mentors meet with mentees on a monthly basis, informal monitoring of OT competency can occur. If required, action can be taken to addresses issues of concern identified by mentor or mentee. After 12 months of delivering the intervention RETAKE OTs competence will be reassessed by evaluating the intervention delivered in a random selection of completed intervention case records (one participant per RETAKE OT) against the trainer's expert opinion. The trainer will review the selected case records against the intervention mechanisms identified in the logic model and confirm whether the intervention delivered is consistent with the intervention that would have been delivered by the trainer as an expert return to work related occupational therapy.

For peer review only - http://bmjopen.bmj.com/site/about/guidelines.xhtml

Fidelity Checklist

A fidelity checklist based on the RETAKE intervention logic model (see Figure 1) and RETAKE intervention process and components will be applied to complete case records (Content of Intervention CRFs, RETAKE OT case notes and Initial Session CRFs) from a random selection of stroke participants randomised to receive the RETAKE intervention (one per treating RETAKE OT). This will be used in measuring adherence to the RETAKE process and identifying factors affecting adherence.

Table 3. CFIF led data extraction for Fidelity Assessment:

**Fidelity Measure**	**CFIF Construct***	**Measurement tool**	**Data for extraction**	**Time point**
Frequency<br><br>Duration	**Adherence and moderating factors**	**Initial Session Case Report Forms (CRFs)**<br><br>**Participant Summary CRFs**	Intervention start date and end date<br>Number of proposed and attended sessions<br>Whether there was an agreed ending for OT return to work support.	One CRF per participant at Initial session.<br><br>One CRF per participant completed throughout intervention delivery

For peer review only

Intensity (time spent per session)<br>Dose (number of sessions)	**Adherence**	**Intervention content CRF**<br><br>**OT clinical records (RETAKE+ Usual Care)**	Time spent (in minutes) on VR activities per session<br><br>Description of intervention delivered in each session	One completed following every intervention session<br><br>In case study participants.
Therapist adherence<br>Factors affecting adherence	**Adherence and moderating factors**	**Fidelity Checklist**	Components delivered, factors affecting delivery<br>RETAKE process followed Y/N	Applied to one randomly selected completed case per RETAKE OT
Real time therapist adherence<br>Factors affecting adherence	**Adherence and moderating factors**	**Mentoring CRFs**	Mentor's concerns about adherence<br>Factors affecting intervention delivery<br><br>Potential solutions	Completed monthly by mentors
Barriers and enablers to intervention delivery	**Moderating factors**	**Interviews with RETAKE Therapists**	Factors affecting intervention delivery<br><br>Potential solutions (developed by OT)	In a random selection of cases during intervention delivery at 3, 6 and 12 months
Acceptability of the intervention<br>Barriers and enablers to intervention delivery	**Moderating factors**	**Interviews with stroke participants, carers, employers and NHS staff**	Acceptability of intervention<br>Factors affecting delivery<br>Potential solutions to barriers	Throughout intervention delivery in case studies

Key; **CFIF Adherence includes intervention content, dose, coverage, frequency and duration of intervention; CFIF Moderating factors includes participant*

For peer review only

For peer review only - http://bmjopen.bmj.com/site/about/guidelines.xhtml

responsiveness, intervention complexity, strategies to facilitate implementation, quality of delivery, recruitment, and context.

Mentor interviews and records

Mentoring records

Following training, each treating OT will be assigned a mentor with extensive knowledge and experience of vocational rehabilitation. Mentoring will take place monthly via teleconference in small groups (four to six therapists) and serve as an intervention implementation support mechanism. RETAKE OTs will be able to discuss any difficulties they are experiencing, ask questions and share best practice with other OTs and their mentor. This process will also facilitate communication between the trial team and enable barriers to implementation and contamination risks to be reported. Key discussion points will be recorded by mentors using a mentoring record form for each session. These records, along with all email correspondence between mentor and mentees will be collected for qualitative content analysis.

Mentor Interviews

Semi-structured interviews will be conducted by two research assistants (SC and KC) with all mentors (n=6) to explore their experiences of supporting RETAKE OTs to deliver the intervention, and ascertain their views of organisational, social and other factors contributing to or affecting delivery of the intervention.

For peer review only - http://bmjopen.bmj.com/site/about/guidelines.xhtml

Social and structural context

Site survey

To describe participating sites and identify potential contaminants, sites will be asked to complete a questionnaire by telephone at three time points; prior to recruitment, halfway through, and at the end of the intervention period. This will contribute to understanding contextual influences through capturing data on existing stroke care pathways and resources (including staff and services) available for supporting participants in a return to work. It will also identify potential contamination risks associated with proposed or planned VR service developments or changes in practice that may influence trial outcomes.

Therapist training

Non-participant observations

To understand OT's experiences of being trained to deliver the intervention, a research assistant (RC) will observe up to four training sessions delivered by the training team. A checklist will be developed using NPT constructs to guide observations. Non-participant observations aim to identify; whether therapists understand the intervention and their role in implementation, whether they think the RETAKE intervention can be integrated into existing practice and any contextual factors affecting the trial.

To describe adherence to the intervention, a researcher will observe up to three sessions for each case study participant in the intervention and usual care arms of the trial. Non-participant observations will be conducted using prompts for structured

For peer review only - http://bmjopen.bmj.com/site/about/guidelines.xhtml

observation and unstructured field notes.[26] Participant selection for inclusion the case study element is described below.

Interviews with Occupational Therapists

Semi-structured interviews will be conducted by a research assistant (RC) with a minimum of one OT per site following their initial RETAKE training to explore their experience of training, the mentoring process and their confidence in intervention delivery. OT's views of the intervention, barriers and facilitators to implementation, and any organisational or social factors impacting on delivery will also be explored. Interviews will take place following training and be repeated at two additional time-points: mid-way through the RETAKE intervention delivery and at the end of the study.

Case studies

Longitudinal case studies will be used to map the care received by RETAKE and usual care participants to develop a more detailed understanding of participants' (stroke survivors, carers, employers) and RETAKE OTs experiences of support for RTW. A 5% subset of participants from both arms of the trial (total n=38) will be randomly selected and invited to participate in the case study element of the process evaluation.

i) Case study interviews

Semi-structured interviews will be conducted by two research assistants (SC and KC) with case study participants at three time points: three, six-, and twelve-months

For peer review only - http://bmjopen.bmj.com/site/about/guidelines.xhtml

post-randomisation, about their experiences and views of and adherence to the RETAKE intervention and the support they received to return to work. The case study participants' carers (if nominated), their employers (where participant consent is obtained) and the OTs providing support for RTW will be interviewed.

NHS staff interviews

To further understand the social and structural factors which influence the implementation of the intervention, interviews will be conducted with up to two (n=34 in total) NHS staff involved in the management, commissioning, or delivery of stroke rehabilitation within each trial site. Participating staff will be chosen using a mixture of purposive and snowball sampling. This will be based on a full range of trial sites, staff knowledgeable about the implementation of the intervention at their site, and staff knowledgeable about the decision-making process relating to wider roll-out.

Additional participant interviews

An additional random 5% of study participants will be invited to participate in semi-structured interviews at the end of the intervention period. These interviews will explore participants' experience of the intervention as well as their perceptions and experiences of returning to work.

All qualitative interviews will be conducted using a topic guide informed by NPT. Examples of question topics and how they relate to the four NPT constructs are shown in Table 1. Topic guides will be presented to the RETAKE Public and Patient Involvement (PPI) group for comment prior to use. All interviews will be audio recorded and transcribed in full.

For peer review only - http://bmjopen.bmj.com/site/about/guidelines.xhtml

Data Analysis

Quantitative analysis

The dose, duration and frequency of the ESSVR intervention will be calculated using data from completed CRFs in combination with NHS therapy records. The total time spent delivering the ESSVR intervention (face to face and non-face to face contact (liaison with the patient, employer and other stakeholders by letter/phone), administration and travel) will be identified. Details relating to the content of intervention sessions will be extracted to identify whether core components of ESSVR were delivered as intended (i.e., as specified in the intervention manual and logic model). Associations between therapist attributes, contextual factors and intervention fidelity (measured by deviations from the RETAKE core process) will be explored using regression models. Analysis will be conducted using Statistical Package for the Social Sciences (SPSS) (version 21.0 for Windows).

Describing Usual Care

Data regarding rehabilitation delivered in Usual Care will be extracted from resource use data in the follow-up questionnaires and from NHS Therapy records in case study participants randomised to Usual Care. These data will be used to inform the cost of Usual Care for the economic evaluation and describe and understand usual care provided during stroke rehabilitation in inpatient and community services. Quantitative analysis of these data will be conducted using Statistical Package for the Social Sciences (SPSS; Version 21.0 for Windows). Analysis of usual care data obtained from NHS Therapy records is described below.

For peer review only - http://bmjopen.bmj.com/site/about/guidelines.xhtml

Qualitative analysis

Inductive (thematic analysis) and deductive (informed by NPT) approaches will be used to guide data analysis and interpretation. Observational and Interview data will be transcribed verbatim and uploaded into QSR NVivo software for management. Descriptions of usual care in NHS Therapy records, observational field note data, including researcher reflections and interview data will be analysed thematically.[27] Framework analysis will be used with the case study data. For each participant the interview data will be coded in NVivo and then imported into a Framework matrix for comparison both within the individual case (comparing views of stroke survivor, carer, OT and employer) and across cases and sites. Analysis will proceed iteratively with data collection to determine whether data saturation has been achieved; researchers will draw on the RETAKE logic model (Figure 1). Throughout the qualitative analysis, NPT will be used as a sensitising framework.

Analysis of each qualitative data set will be conducted independently and then jointly by at least two study team members (SC, KC, KP) to corroborate themes and discuss any discrepancies. It will follow a standard inductive approach of data familiarisation, line-by-line coding and development of broad themes. Themes will then be mapped to NPT constructs as part of development and refinement of broader conceptual explanatory categories. Researchers will keep a set of interim summary notes documenting any reflexivity points and connections between the data with NPT and the logic model, to aid analytical discussions with the wider process evaluation team. Iterative testing of interpretation will occur through discussion with and feedback from the PPI group and discussions within the research team.

For peer review only - http://bmjopen.bmj.com/site/about/guidelines.xhtml

Synthesis of qualitative and quantitative data

During the RETAKE trial the qualitative and quantitative data generated as part of the process evaluation will be independently analysed by the process evaluation team and the Clinical Trials Research Unit respectively. Data related to intervention fidelity and description of usual care will be synthesised at the conclusion of the trial. We will review and compare findings from related data sets, identify areas of agreement and disagreement and develop explanations for the findings. Synthesis of findings from both the quantitative and qualitative data generated will contribute directly to the overall evaluation and explanation of the outcomes of the RETAKE trial.

Ethics and dissemination

Ethics approval has been obtained through the East Midlands – Nottingham 2 Research Ethics Committee (REC) (Ref: 18/EM/0019) and the National Health Service Research Authority. The procedures for gaining informed consent have been detailed above. Dissemination will be via journal publications, stroke and rehabilitation focused conferences, newsletter articles, social media, presentations to clinicians and stroke survivors and meetings with national clinical leads for the Stroke Plan and the NHS Plan.

Availability of data and materials

No additional data will be made available.

For peer review only - http://bmjopen.bmj.com/site/about/guidelines.xhtml

Competing interests

The authors declare that they have no competing interests.

Funding

This study is funded by the NIHR HTA programme (ref: 15/130/11). The views expressed herein are those of the authors, not necessarily the NIHR, the Department of Health and Social Care, or the NHS.

Authors' contributions

KR, CM, AFa, AB, ROC, MW, and CW conceived the study. KR, DJC, and CM designed the process evaluation. KR, CM, DJC, SC, KC, JH, JP, TS, RC and KP operationalized the process evaluation protocol. KR, JP, and JH designed the intervention. AS has the role of trial sponsor. IH, RB, and AFa devised the data management and statistical analysis plan. JS and JM acted as PPI collaborators to support plans for trial design/delivery, management, and dissemination of trial findings. VM and SH have responsibility for management of the trial. KR, SC and DJC drafted the manuscript; all other authors read and approved the final version.

For peer review only - http://bmjopen.bmj.com/site/about/guidelines.xhtml

REFERENCES

1. Stroke Association. State of the Nation: stroke statistics. Stroke Association. 2018.https://www.stroke.org.uk/resources/state-nation-stroke-statistics (accessed 27 Apr 2021).
2. Daniel K, Wolfe CD, Busch MA, et al. What are the social consequences of stroke for working-aged adults? A systematic review. *Stroke*2009;40:e431-40.
3. Sinclair E, Radford K, Grant M, et al. Developing stroke-specific vocational rehabilitation: a soft systems analysis of current service provision. *Disabil Rehabil* 2014;36:409-17.
4. Corr S, Wilmer S. Returning to work after a stroke: an important but neglected area. *Br J Occup Ther* 2003;66:186-92.
5. Frank AO, Thurgood J. Vocational rehabilitation in the UK: opportunities for health-care professionals. *Int J Ther Rehabil* 2006;13:126-34.
6. Lindström B, Röding J, Sundelin G. Positive attitudes and preserved high level of motor performance are important factors for return to work in younger persons after stroke: a national survey. *J Rehabil Med* 2009;41:714-8.
7. Palstam A, Westerlind E, Persson HC, et al. (2019). Work-related predictors for return to work after stroke. *Acta Neurol Scand* 2019;139:382-388. doi:10.1111/ane.13067

For peer review only - http://bmjopen.bmj.com/site/about/guidelines.xhtml

8. Waddell G, Burton AK, Kendall NA. Vocational rehabilitation–what works, for whom, and when? (Report for the Vocational Rehabilitation Task Group). London: TSO 2008.

9. Escorpizo R, Reneman MF, Ekholm J, et al. A conceptual definition of vocational rehabilitation based on the ICF: building a shared global model. *J Occup Rehabil* 2011;21:126-33.

10. Ntsiea MV, Van Aswegen H, Lord S, et al. The effect of a workplace intervention programme on return to work after stroke: a randomised controlled trial. *Clin Rehabil*2015;29:663-73.

11. Trexler LE, Trexler LC, Malec JF, et al. Prospective randomized controlled trial of resource facilitation on community participation and vocational outcome following brain injury. *J Head Trauma Rehabil*2010;25:440-6.

12. Radford K, King K, McLellan V, et al. An individually randomised controlled multi-centre pragmatic trial with embedded economic and process evaluations of early vocational rehabilitation compared with usual care for stroke survivors: study protocol for the RETurn to work After stroKE (RETAKE) trial. Submitted to Trials 2020.

13. Grant M, Radford K, Sinclair E, Walker M. Return to work after stroke: recording, measuring and describing occupational therapy intervention, British Journal of Occupational Therapy, 2014; 77(9) 457-465. https://doi.org/10.4276/030802214X14098207541072

14. Lynch EA, Chesworth BM, Connell LA. Implementation—The missing link in the research translation pipeline: is it any wonder no one ever implements evidence-based practice?. *Neurorehabil Neural Repair*2018;32:751-61.

For peer review only - http://bmjopen.bmj.com/site/about/guidelines.xhtml

15. Glasziou P, Straus S, Brownlee S, et al. Evidence for underuse of effective medical services around the world. *Lancet* 2017;390:169-177. doi:10.1016/S0140-6736(16)30946-1
16. Walker MF, Hoffmann TC, Brady MC, et al. Improving the development, monitoring and reporting of stroke rehabilitation research: Consensus-based core recommendations from the Stroke Recovery and Rehabilitation Roundtable. *Int J Stroke* 2017;12:472-9.
17. Moore GF, Audrey S, Barker M, et al. Process evaluation of complex interventions: Medical Research Council guidance. *BMJ*2015;350:h1258.
18. Basch CE, Sliepcevich EM, Gold RS, et al. Avoiding type III errors in health education program evaluations: a case study. *Health Educ Q* 1985;12:315-31.
19. Dusenbury L, Brannigan R, Falco M, et al. A review of research on fidelity of implementation: implications for drug abuse prevention in school settings. *Health Educ Res* 2003;18:237-56.
20. Colditz GA, Emmons KM. The promise and challenges of dissemination and implementation research. *Dissemination and implementation research in health: Translating science to practice*2012;24:3-22.
21. Bosch M, VanDerWeijden T, Wensing M, et al. Tailoring quality improvement interventions to identified barriers: a multiple case analysis. *J Eval Clin Pract*2007;13:161-8.
22. Levac D, Glegg SM, Sveistrup H, et al. A knowledge translation intervention to enhance clinical application of a virtual reality system in stroke rehabilitation. *BMC Health Serv Re*s2016;16:557.
23. Ioannidis JP, Greenland S, Hlatky MA, et al. Increasing value and reducing waste in research design, conduct, and analysis. *Lancet* 2014;383:166-75.

For peer review only - http://bmjopen.bmj.com/site/about/guidelines.xhtml

24. May C, Murray E, Finch T, et al. Normalization process theory online users' manual and toolkit. NPT: Normalization Process Theory. 2010. http://www.normalizationprocess.org/ (accessed 27 Apr 2021).

25. Carroll C, Patterson, M, Wood, S, et al. A conceptual framework for implementation fidelity. *Implementation Sci*2007;2:40.

26. Ciesielska M, Boström KW, Öhlander M. Observational methods. In: Ciesielska M, ed. Qualitative Methodologies in Organization Studies Volume II: Methods and Possibilities. Cham, Switzerland. Springer 2018:33-52.

27. Braun V, Clarke V. Using thematic analysis in psychology. *Qual Res Psychol* 2006;3:77-101.

For peer review only - http://bmjopen.bmj.com/site/about/guidelines.xhtml

Early Stroke Specialist Vocational Rehabilitation (ESSVR) Logic Model
Aim: To support patients who have had a stroke to return to and remain in work.

Rationale for ESSVR

The problem:
-Return to work (RTW) is achieved by less than 50% of stroke survivors
-Work is essential for supporting health, wellbeing, and longevity
-Long-term unemployment linked to increased risk of depression, suicide, reduced quality of life, cardiovascular disease, and health-harming behaviours
-Vocational rehabilitation (VR) supports those disadvantaged by illness or disability to access, return to, and maintain employment or another useful occupation

Resources

Resources required and context for intervention delivery:
-Skilled OT, knowledgeable in stroke. Trained in ESSVR
-Experienced mentor support for OTs delivering ESSVR
-Stroke ward staff identify all patients employed at time of stroke & refer to VR OT
-Effective co-location - crossing boundaries between health, employment, III sector
-Stroke patient wants to work
-Supportive employer

RTW intervention

Core ESSVR components and mechanisms:
-VR OT **intervenes early** ≤8 weeks of stroke (gives early advice on impact of stroke & RTW to patient and healthcare professional)
-**Assesses** impact of stroke on person/family & job (analysis of work ability, worksite assessment)
-Delivers **individually tailored** VR (work preparation, RTW planning)
-**Communicates** openly in writing with stakeholders re work status
-**Coordinates** VR across all sectors
-**Provides education**, advice & emotional support to patient, family & employer
-**Mediates workplace adjustments**, negotiates phased RTW, provides feedback on performance
-**Monitors RTW** to ensure work sustainability (regular review, employer supported to provide feedback on work performance, feedback on progress and modification)
-**Explores alternatives** where current work cannot be sustained/is not feasible
-**Gradual withdrawal** of intervention, which patient can re-access as required

Individual, organisational and system linked outcomes

**Individual outcomes:**	**Organisational outcomes:**	**System outcomes:**
-Health supported by being in good work<br>-Patient satisfied with decisions made about work<br>-Patient & employer satisfied with intervention<br>-Patient & employer feel supported in job retention<br>-Patient reports increased stroke confidence<br>-Patient reports improved self-efficacy in managing at work	-Prevent job loss<br>-Increased opportunities for employer engagement by intervening early<br>-Work & workplace is appropriate for patient<br>-Workplace adjustments & strategies in place<br>-Optimised productivity at work by patient	-Reduced health resource usage<br>-Contributes to economy<br>-Reduced Welfare benefits use

Trial outcome

Trial outcome measurement:
-EQ5D.5L, CaSM, HADS, NEADL, CIQ, Work Ability Index (1Question)
-Self Report RTW at 12 months
-RTW same employer (3,6,12 months)
-Days and -Hours worked (3,6,12 months)
-Process Evaluation including fidelity, NPT informed individual case studies, participant interviews (intervention and usual care), staff interviews.

Moderating and contextual factors: Legal employment framework; National Clinical Guidelines for Stroke; Clinical Commissioning for stroke services; Cross sector engagement in VR programme support; Stroke survivor support and social network.

For peer review only - http://bmjopen.bmj.com/site/about/guidelines.xhtml

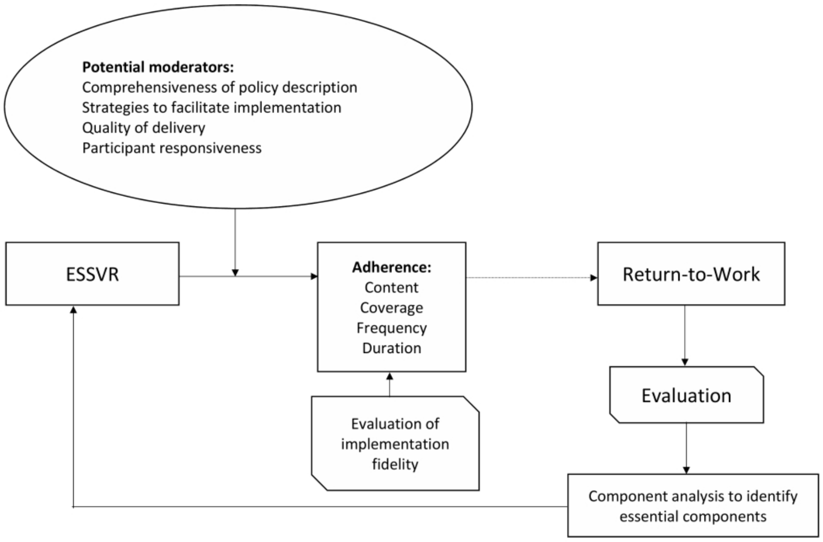

Figure 2: Assessment of fidelity and factors moderating ESSVR delivery in accordance with the Conceptual Framework for Implementation Fidelity

297x210mm (300 x 300 DPI)

Appendix I	**ESSVR Description (TIDieR)**
**Brief Name** (Provide the name or a phrase that describes the intervention.)	1a) Early Stroke Specific Vocational Rehabilitation (ESSVR)<br>1b) The Return to Work after Stroke (RETAKE) trial
**WHY** Describe any rationale, theory, or goal of the elements essential to the intervention.	**Rationale**<br><br>Stroke is common (>100,000 strokes per annum in the UK) [1]. In spite of reperfusion therapy and secondary prevention, outcomes remain poor - almost two-thirds of survivors leave hospital with a disability, and a third experience depression and/or cognitive impairment. Stroke survivors of working age are 2-3 times more likely to be unemployed [1].<br><br>Increasingly, there is an expectation that existing health and social care pathways for stroke survivors provide support for stroke patients intending to return to work [2-9]. Despite improvements in the organisation of stroke rehabilitation services following discharge, many stroke survivors fail to access this support because a) their work rehabilitation needs are not identified early after stroke b) many have hidden disabilities such as visual or cognitive impairments and fatigue, which are missed in the acute phase [10] and c) the criteria for referral to community rehabilitation are impairment based rather than needs led, meaning that a person with unmet needs for work participation alone (rather than a need for support from more than one healthcare professional e.g. Occupational Therapy and Speech and Language Therapy) may be unable to access support. d) Not all community stroke services provide rehabilitation that addresses work needs [11]. Where they do this may be time limited or fail to engage with employers in the workplace, as supporting a return to work is not always seen as the job of health [9]. Furthermore, stroke survivors themselves may not appreciate the true impact of the stroke on their workability until they attempt to return to work [12].<br><br>Failure to provide this support, may lead to job loss, affecting physical, emotional, and financial wellbeing and quality of life [13,14]. Return to work is a recognised outcome of health interventions [15]. Supporting people who develop health conditions to return to work is recommended in stroke policy and clinical guidelines [3,4,5,7].<br><br>The UK government has committed to reduce the employment gap (54% Vs 82%) between disabled and non-disabled people. Its goal is to see one million more disabled people in work by 2027 [16].<br><br>The Equality Act requires employers to make reasonable adjustments, to accommodate the person in the workplace [17]. These adjustments may involve more breaks, reductions in working hours, reduced responsibilities, increased supervision, flexible working patterns and working from home and help from other people or agencies, including rehabilitation.<br><br>**The 'theory of change underpinning ESSVR'**<br><br>Health based preparation and support for returning to work after stroke has typically been deficient in meeting stroke survivors work needs. ESSVR was designed to bridge the gap between existing stroke rehabilitation services, the employment and the voluntary sector in supporting stroke survivors in a return to work [10] Tested in a single centre feasibility trial we found evidence to suggest that that the intervention may have potential to support job retention at 12 months post stroke [18].<br><br>The implicit theory of change on which ESSVR can be expressed as follows:

For peer review only

	Stroke brings about physical and psychological impairments that are likely impact on the capacity to return to and remain in work<br><br>The ability to identify work needs early in the stroke pathway is missing from stroke services and vocational rehabilitation knowledge and skills gap is present in stroke rehabilitation services. Implementing mechanisms for identifying stroke survivors who are employed at stroke onset; educating the stroke care team about 'return to work' and teaching OTs with stroke specific knowledge basic skills in vocational rehabilitation, disability discrimination, how to evaluate jobs and assess work capability and match stroke survivor's abilities to job demands;<br>how to engage with employers, and other employment sector stakeholders, to go into the workplace and how to negotiate reasonable adjustment and phased return to work will enable stroke services to support stroke survivors in a return to work.<br><br>The logic model (Figure 1) has the following underlying assumptions;<br><br>• *If* we implement an early 'VR pathway' for stroke *then,* work is seen as a health outcome by stroke rehabilitation teams, conflicting advice prevented, increased confidence, knowledge and skills in VR, patient aware of available support & how to access; Early barriers to RTW identified e.g. environmental (job type), personal. Recognising work as an outcome of health interventions thus promoting a shared philosophy of rehabilitation to support return to work **[Mechanism: Early Intervention, Collective Understanding]**<br><br>• *If* we identify people who are employed at the time of stroke and refer to an Occupational Therapist trained in VR (VR OT) for information/advice/ support re return to work (RTW), *then* this will increase opportunities for RTW & prevent job loss; prevent people from falling into service gaps, and ensure work needs are met. [**Mechanism: Early Identification]**<br><br>• *If we* teach OTs basic skills in vocational rehabilitation (how to evaluate jobs and assess work capability, match the injury related disabilities to job demands; how to engage with employers, and other employment sector stakeholders, go into the workplace and how to negotiate reasonable adjustment and a phased return to work) *then* they will have the confidence, knowledge and skills to support stroke survivors in a return to work **[Mechanism: VR Upskilling; Clinicians confident and empowered; Assessment]**<br><br>• *If the OT* provides early (within 8 weeks of stroke) assessment, education and advice on the impact of stroke & RTW, *then* the impact of the stroke on the job role will be identified to inform a vocational rehabilitation plan. Persons requiring psychological support for mental health issues are identified and referred for support, resulting in improved physical and mental health and financial wellbeing. **[Mechanisms: Assessment; Education Early intervention]**<br><br>• *If the OT* delivers individually tailored vocational rehabilitation, engaging with the employer to negotiate workplace accommodations, a phased return to work, educating employers and monitors ongoing work ability, *then,* the person will be able to cope with work, resulting in reduced sickness absence and sustainable employment. **[Mechanisms: Individual Tailoring; Accommodating stroke at work, Colocation, Employer Engagement, communication]**<br><br>ESSVR is a biopsychosocial intervention informed by the International Classification of Function (ICF) [19] and the 'Work Disability Arena' or Sherbrooke model [20]. It takes into consideration the overall context of an individual. It identifies the level of functioning at the body, person and societal level, as well as understanding the personal and environmental contextual factors that may impede or enhance work participation.

For peer review only

	It aims to prevent job loss by drawing on employment law and the Equality Act (2010) (17) to prevent disability discrimination and ensure "reasonable adjustments" are negotiated with employers to reduce the impact of stroke disability by accommodating (modifying) the stroke survivor's job to enable a return to work. ESSVR also ensures patients are provided with appropriate individualised work-related physical and cognitive rehabilitation and self-management education to increase their ability to work.
**WHAT**<br><br>**Materials**: Describe any physical or informational materials used in the intervention, including those provided to participants or used in intervention delivery or in training of intervention providers. Provide information on where the materials can be accessed (e.g. online appendix, URL).<br><br>**Procedures**: Describe each of the procedures, activities, and/or processes used in the intervention, including any enabling or support activities.	**Materials:**<br><br>**Training:** Occupational therapists are provided with an 'ESSVR Intervention manual' detailing the intervention content, its rationale and objectives, processes to be followed and forms for use in documenting ESSVR delivery in the trial. The manual included examples of return to work plans, sample graded RTW planning, session and work review letters, sample letters to GP, discharge letters, letter to employer, sample report for occupational health and a list of other useful resources (below). The manual was sent to therapist two weeks before the training and used during the training to navigate them through the ESSVR intervention process and familiarise them with its contents and resources.<br><br>**Resources included:**<br><br>For Occupational Therapists<br><br>• Employment and Support Allowance (ESA) Supporting letter and Guide to completing ESA (2012), See 50 9 esa50guide2012 (nawra.org.uk)<br>• Allied Health Professions Fitness For Work Report (RCOT), Accessible via https://www.rcot.co.uk/practice-resources/standards-and-ethics/ahp-health-and-work-report<br>• AHP Health and Work Report: Guidance for AHP practitioners on the use and completion of the Report (Allied health Professions Federation). See; Guidance-on-completion-of-AHP-Health-and-Work-Report.pdf (ahpf.org.uk)<br>• Graded RTW planning leaflet (RETAKE Trial specific)<br>• Tailored Adjustments Plan (Business Disability Forum, 2020) Accessible via Tailored Adjustments Plans - Business Disability Forum<br>• Work Ability Support Scale (WSS) (Fadyl J, McPherson KM, Schulter P, Turner-Stokes L., 2014) [21] Accessible via https://www.kcl.ac.uk/cicelysaunders/resources/tools/wss<br>• WSS Detailed work questionnaire, Accessible via https://www.kcl.ac.uk/cicelysaunders/resources/tools/wss<br>• WSS Brief work questionnaire and jobe matching, Accessible via https://www.kcl.ac.uk/cicelysaunders/resources/tools/wss<br>• THE CITY OF TORONTO S JOB DEMANDS ANALYSIS AND JOB MATCH SYSTEM (Lucas, 2017), accessible via; https://silo.tips/download/the-city-of-toronto-s-job-demands-analysis-and-job-match-system<br>• Beginners Guide to Benefits, Accessible via https://www.turn2us.org.uk/Benefit-guides/Beginner-s-Guide-to-Benefits/Checking-benefit-entitlement<br>• Good work for good health The difference occupational therapy makes, (RCOT, 2019) Accessible via ILSM Work report A4 7pp D7.pdf (rcot.co.uk)<br><br>For Employers<br><br>• Employees with Executive Functioning Deficits (Job Accommodation Network 2018) , Accessible via; Brain Injury (askjan.org)<br>• Accommodation and Compliance Series: Employees with Speech-Language Impairment (Job Accommodations Network, 2019) Accessible via JAN-Job-accomadation-suggestions.pdf (dysphonia.org)

	• Job accommodations for people with motor limitations from stroke (Morgantown, WV, Office of Disability Employment Policy, Job Accommodation Network, 2010) Accessible via Job accommodations for people with motor limitations from stroke - University of Missouri Libraries<br>• A complete guide to stroke for Employers (Stroke Association, 2019), See: f41cg_a_complete_guide_to_stroke_for_employers_v3_oct_2019.pdf,<br>• Information Pack -Work After Stroke - Information for Employers, (Different strokes, 2018) Available at: Work After Stroke (differentstrokes.co.uk)<br><br><u>For stroke survivors</u><br><br>• Information Pack Work After Stroke - Information for Family & Friends (Different Strokes, xxx year) Accessible via: Work After Stroke - Information for Family & Friends<br>• A_complete_guide_to_work_and_stroke.pdf See: Your rights at work after stroke | Stroke Association, (Stroke Association, UK)<br>• Driving after a Stoke guide; (Stroke Association, 2021) See f02_driving_v_3.1_web_june_21.pdf (stroke.org.uk)<br>• Stroke in people of working age (Stroke Association, 2014), Accessible via: stroke_in_people_of_working_age.pdf<br>• Tailored Adjustments Plan (Business Disability Forum, 2020) Accessible via Tailored Adjustments Plans - Business Disability Forum<br><br>**Links provided to other Online Resources**<br><br>**Advisory services**<br>• ACAS- Advisory, Conciliation and Arbitration Service- provides support in assisting employment disputes including those related to disability management: http://www.acas.org.uk<br>• Citizens Advice Bureau: http://www.citizensadvice.org.uk/<br>• Disability Law Service: www.dls.org.uk<br>• Disability Rights UK http://disabilityrightsuk.org/<br>• Equality and Human Rights Commission http://www.equalityhumanrights.com/<br><br>• **Occupational Health Advisory Service** – Fit for Work offers free, expert and impartial advice to anyone looking for help with issues around health and work. You can browse our online resources, chat online to a specialist advisor, email a question or call our free advice line on 0800 032 6235 (English) or 0800 032 6233 (Cymraeg). https://fitforwork.org/<br><br>**Details of occupational health providers**<br>• Occupational health support can be very helpful in complex cases Occupational health services are sometimes provided by NHS or local authority services. To find details of providers in your area, contact:<br>• Commercial Occupational Health Provider Association www.cohpa.co.uk<br>• NHS Health at Work www.nhshealthatwork.co.uk/support-for-business.asp<br>• Society of Occupational Medicine www.som.org.uk<br>• Safe Effective Quality Occupational Health Service (list of approved occupational health providers) http://www.seqohs.org<br><br>**Job Centre Plus:**<br>• Disability Employment Advisers are based in Jobcentres, and work with claimants facing complex employment situations because of a disability or health condition. They can act as an advocate with prospective employers if necessary, aiming to identify work solutions that will overcome or minimise any difficulties related to an individual's disability in the work place. https://www.gov.uk/specialist-employability-support<br>• Welfare Benefits and Department for work and Pensions (DWP)<br>• Benefits (including Attendance Allowance, Employment Support Allowance, and Disability Living Allowance/Personal Independence Payment): https://www.gov.uk/browse/disabilities/benefits

- Access to Work information including contact details for all centres (for registration, the initial step for clients wanting to use this scheme): https://www.gov.uk/access-to-work/overview
- Benefits and Work website offers advice to people re benefits. Some free information, fee for access to additional support http://www.benefitsandwork.co.uk/

Debt issues

- https://www.citizensadvice.org.uk/debt-and-money/
- https://www.nationaldebtline.org/
- http://www.debtadvicefoundation.org/

Equipment advice:

- A huge range of IT accessibility info, assessments, resources: http://www.abilitynet.org.uk/
- Disabled Living Foundation: http://ww.dlf.org.uk

Guidelines:

- Vocational Rehabilitaiton Associaiton Guidelines- free to download upon registration: https://vrassociationuk.com/
- BSRM Publications free to download- VR and long term conditions; VR Interagency guidelines:
- https://www.bsrm.org.uk/publications/publications

Fit Note

- AHP Fitness to Work Report info: http://www.ahpf.org.uk/AHP_Advisory_Fitness_for_Work_Report.htm
- Fit Note info: https://www.gov.uk/government/collections/fit-note

- Managing sickness absence, disputes and sick pay
- Gov.uk - https://www.gov.uk/employers-sick-pay

The Health and Safety Executive has provided guidance for employers and managers on managing sickness absence and return to work.

- www.hse.gov.uk/pubns/priced/hsg249.pdf

British Occupational Health Research Foundation has also developed guidance for managing sickness absence and return to work. www.bohrf.org.uk/downloads/Managing_Rehabilitation-Guidance.pdf

For questions about **Statutory Sick Pay** you can visit the HMRC website at https://www.gov.uk/topic/business-tax/paye or call them on 08457 143143.

The Employer's Charter helps employers understand what they can do in respect of a number of issues.
www.gov.uk/government/uploads/system/uploads/attachment_data/file/32147/employerscharter.pdf

- Touchbase: DWP news about work, working age benefits, pensions and services (DWP, 2015) Accessible via: Touchbase: DWP news about work, working age benefits, pensions and services - GOV.UK (www.gov.uk)

Job search:

- https://www.gov.uk/jobsearch
- http://www.indeed.co.uk
- https://jobs.civilservice.gov.uk/company/nghr/jobs.cgi
- http://jobs.theguardian.com/
- http://www.jobs.nhs.uk/
- http://www.charityjob.co.uk/
- http://www.jobhuntersbible.com/
- http://www.jobsgopublic.com/searches/new

	**Stroke information**<br>• Different strokes - https://differentstrokes.co.uk/ (for younger stroke pts)<br>• Stroke association https://www.stroke.org.uk<br><br>• **VR general:**<br>• MS Trust/Society and Headway - links to toolkits<br>• Job Accommodation Network https://askjan.org/<br>• British Association of Supported Employment http://base-uk.org/<br><br>• **Volunteering associations**<br>• https://www.ncvo.org.uk/ncvo-volunteering<br>• https://do-it.org/<br><br>**Fitness/health information** http://www.nhs.uk/Livewell/fitness/Pages/free-fitness.aspx<br>• Cinema Exhibitor card https://www.cinemauk.org.uk/key-issues/disability-and-access/cea-card/<br>• If a person gets DLA, PIP or is registered blind, they can get this card and it entitles a free entry for another person<br>• Local walk for health schemes http://www.walkingforhealth.org.uk/walkfinder/ -<br><br>**Transport**<br>• DVLA (driver vehicle licencing authority)<br>• https://www.gov.uk/stroke-and-driving (patient information)<br>• https://www.gov.uk/current-medical-guidelines-dvla-guidance-for-professionals<br><br>**Disabled bus pass**<br>• If not allowed to drive for a year due to their injury, they are entitled to a disabled bus pass<br>• https://www.gov.uk/apply-for-disabled-bus-pass<br><br>• **Goal Attainment Scaling (GAS)** in Rehabilitation system https://www.kcl.ac.uk/cicelysaunders/resources/tools/gas<br><br>**Procedures:**<br><br>***Intervention Delivery***<br>ESSVR is an early, individually tailored, stroke specific job retention intervention. It adopts a problem- solving process, which involves vocational goal setting and regular progress review. It aims to adapt the environment and accommodate the stroke survivor at work. It also aims to educate the person to self-manage the condition at work.<br>It involves a trained vocational rehabilitation OT adopting a role as a case coordinator with a wider team of healthcare professionals, employers, family members and other agencies (e.g. occupational health and employment services, GPs, independent and voluntary sector services) to:<br><br>• Assess the impact of the stroke on the patient, family and the patient's role as a worker/student and their ability to do their job/study course.<br>• Educate participants, employers/tutors and families about the effects of stroke and its impact on work/education and find acceptable strategies to lessen the impact.<br>• Monitor and assess the patient's work/educational goals.<br>• Prepare people for work/education by establishing structured routines with gradually increased activity levels and opportunity to practice work skills, e.g., structured computerised cognitive stimulation to increase concentration, daily walks to increase physical stamina.<br>• Liaise with employers/tutors, employment advisors, student services and the healthcare team to advise about the effects of stroke and to plan and monitor a phased return to work.<br>• Alternatives to pre-injury employment are explored in cases where return to pre-existing employer is not feasible or unsustainable.

	The Occupational Therapist VR role involves, negotiating workplace accommodations, communicating with employers, offering advice and emotional to the patient, the patient's family and employer, and exploring work alternatives as required. The case-coordination role involves theRETAKE OT actively coordinating the RTW and input from relevant services from across all sectors (health, work, independent, voluntary, education), communicating with all involved stakeholders, such as the participants GP Department for Work and Pensions Services, welfare rights and employer organisations e.g. occupational health, GPs and voluntary sector services e.g. the stroke Association. The aim being to maximise the use of all locally available resources and ensure consistent advice and support for the patient.<br><br>ESSVR is a process (rather than a set of predetermined components) that is broken into 3 stages;<br><br>**Stage 1: Early recovery and Work preparation:** The OT intervenes early, within 8 weeks of stroke onset, to ensure work is on the agenda and jobs are not relinquished but kept open. Assessment of the individual, the impact of the stroke and a detailed job analysis and liaison with family members takes place at this stage. Plans are made to prepare the RETAKE participant for work return by providing advice and information to the participant and their family and advise medical/other rehab staff to encourage the participant not to make immediate decisions about work i.e. leaving work or going back too soon, which may jeopardise their RTW or job retention. The RETAKE participant is encouraged to keep the channels of communication with the workplace open and the RETAKE OT offers to mediate if difficulties arise. Activities are undertaken at home, relevant to work or simulated to build up the stamina and skills required to return to specific work tasks or roles. These include physical, cognitive or communication based activities depending on how the stroke has affected the RETAKE participant and the demands of their job. Liaison with any other services the person is receiving takes place to ensure there is no overlap and the approach to VR is smoothly coordinated.<br><br>**Stage 2: Graded return to work:** This involves planning, negotiating and implementing a phased return to work (RTW). This might involve a worksite visit, negotiation of realistic timing and identification of workplace adjustments/accommodations to optimise RTW. Liaison with Human Resources (HR), occupational health, other employer bodies and medical teams may also take place. Information and education is provided for employers to increase their understanding of the impact of the stroke on the RETAKE participant and how this might influence their ability to meet job demands. The participant receives feedback on their work performance during this stage. This may involve regular reviews, feedback on progress and supporting the employer to provide feedback on work performance, and the implementation of any modifications to the RTW plan or work role.<br><br>**Stage 3: Job Retention:** This involves monitoring the participant's RTW to ensure work stability and troubleshooting issues that may arise with all stakeholders (patient, employer, family, others) and gradually withdrawing support when the work situation is stable. However, participants and employers can re-access this support as required up to 12 months post randomisation. In some cases where work cannot be sustained or is unfeasible, work alternatives e.g. voluntary work, changes in job type, career are explored. In some cases the intervention may involve supporting retirement or medical withdrawal from work.<br><br>The intervention is delivered in addition to the stroke participant's usual stroke rehabilitation. This will vary depending on local provision and individual participants' needs. Therefore, the RETAKE OT liaises with health care professionals providing usual stroke rehabilitation to clarify and agree roles and ensure that any vocational rehabilitation is provided by the RETAKE OT.<br><br>The RETAKE OT works in partnership with other health, social care, charitable, employment and independent sector service providers in delivering the ESSVR. Any parallel rehabilitation or other wider services involved (e.g. other OTs, Social Services, Jobcentre Plus, Occupational Health, Different Strokes) are kept informed of the ESSVR process, the RETAKE participant's progress and the RETAKE OTs involvement. RETAKE OTs will refer to, liaise with and help participants to access any service they need, and attend DWP appointments or Occupational Health meetings with participants if required.<br><br>Assessment of the impact of the stroke on the person and the job may involve the use standardised assessments of function and impairment e.g. mobility and cognition, functional capacity evaluation, work needs, and detailed job analysis. Specific tools are not prescribed but rather introduced and resources signposted.

For peer review only

	For more detailed descriptions of the intervention delivered in the feasibility trial see;<br><br>Grant M. (2016) Developing, delivering and evaluating stroke specific vocational rehabilitation: A feasibility randomised controlled trial (Doctoral dissertation, University of Nottingham).<br><br>Grant M, Radford K, Sinclair E, Walker M (2014) Return to work after stroke: recording, measuring, and describing occupational therapy intervention. British Journal of Occupational Therapy, 77(9), 457–465.
**WHO PROVIDED**<br>For each category of intervention provider (e.g. psychologist, nursing assistant), describe their expertise, background and any specific training given.	**Intervention provider qualifications**<br>The intervention was delivered by qualified and HealthCare Professions Council (HCPC) registered occupational therapists (OTs).<br><br>**Intervention provider background and experience**<br>The OTs require experience of working with people with stroke and/or other neurological conditions and community rehabilitation experience. Some may have vocational rehabilitation experience.<br><br>The level of experience and suitability of the therapists recruited to deliver the intervention is assessed by the Chief Investigator and OT mentors prior to training.<br><br>**Training provided**<br>The training comprised 2-days of face-to face teaching delivered by the RETAKE training team (4 OTs experienced in vocational rehabilitation and research) followed by an additional day, 6 months later, supported by monthly small group-based (4-6 OTs) telephone/ videocall mentoring from occupational therapists with extensive experience in delivering vocational rehabilitation following stroke. The OT mentors were members of the training team. Three members of the OT training team held a PhD.<br>The purpose of mentoring is to ensure implementation and fidelity to the intervention process through discussion of difficulties and sharing of best practice with other OTs and their mentor.<br><br>Prior to training, occupational therapists were signposted to papers relating to the RETAKE feasibility trial findings and were sent a RTW case study, which required them to provide written responses to 6 questions and return to the training team prior to training. This enabled the expert trainers to ascertain the OTs pre-training vocational rehabilitation knowledge. The same case study was used to teach the ESSVR process during the training.
**HOW**	**Mode of delivery**<br>The intervention is delivered face-to-face or via telerehabilitation (video call or phone call) on a 1 to 1 basis.<br><br>**Other**<br>Additional time is spent in liaison (letters, phone and video calls) with the patient, employer, family or other stakeholders. Most progress monitoring in stage 3 is delivered by telephone.
**WHERE**	**Where provided**<br>The intervention is delivered in the community (mostly in the home or in the workplace). Other locations may include the meeting room of a disability rights charity (13%), and a voluntary organization jobs brokerage centre (7%). In the feasibility trial almost half of the participants were initially seen in hospital or in a stroke rehabilitation unit.

For peer review only

**WHEN and HOW MUCH.**	**Intervention delivery time**<br>The intervention commences within 8 weeks of stroke and continues for up to 12 months following the initial session. The duration of intervention and frequency of contacts is determined by individual participant's needs. Based on feasibility trial data (Grant, 2014), two thirds of the OTS time will be spent delivering the intervention either face-to-face or in liaison with the participant and others. The other third is spent writing notes and reports or travelling to see participants at home or their work places.<br><br>**Number of sessions and length**<br>Based on feasibility trial data the estimated mean number of face-to face sessions per participant is 10 (SD 7, range 1–25) and average session length is one hour. People with more moderate and severe stroke may require more sessions.<br><br>**Frequency of sessions**<br>More interventions sessions will be delivered at the outset of the intervention during stages 1 and 2 with less frequent interventions in stage 3, during progress monitoring once the participant has RTW.
**TAILORING**<br>If the intervention was planned to be personalised, titrated or adapted, then describe what, why, when, and how.	The ESSVR intervention will be tailored in duration and frequency according to individual need over a 12-month period.
**MODIFICATIONS**	During the current trial intervention delivery continued according to local NHS Trust protocols throughout the COVID-19 pandemic. In some sites OTs continued to visit participants at home wearing personal protective equipment, in others delivery was via telerehabilitation (online or telephone).
**HOW WELL**	**Planned**<br><br>Throughout the trial fidelity to the intervention process will be measured and monitored as described in Table 2 and summarised below.<br><br>Frequency duration and dose will be recorded using case report forms (CRFs), capturing Intervention start date and end date, Number of proposed and attended sessions, Whether there was an agreed ending for OT return to work support; Time spent (in minutes) on VR activities per session and from the description of intervention delivered in OT clinical records.<br><br>Adherence and Factors affecting adherence will be measured using an ESSVR fidelity checklist (Powers, in preparation) and recorded on mentoring CRFs during monthly mentoring sessions led by an experienced vocational rehabilitation OT. implementation barriers and contamination risks will be communicated to the trial team, enabling barriers to be managed in real time.<br><br>Factors affecting intervention delivery will be recorded in Interviews with RETAKE Therapists, participants with stroke, their employers and other NHS staff as part of a series of embedded case studies.
Actual: If intervention adherence or fidelity was assessed,	

For peer review only

describe the extent to which the intervention was delivered as planned.	

References

1. Stroke statistics: sources and definitions | Stroke Association 2021, Accessed 18 September 2021
2. Tyerman A, Meehan M. Vocational assessment and rehabilitation after acquired brain injury: inter-agency guidelines. Royal College of Physicians of London. 2004. https://www.bsrm.org.uk/downloads/vocational-assessment-rehabilitation-abi.pdf. Accessed 06 April 2020.
3. Department of Health (DH). National Stroke Strategy. DH. 2007. https://www.england.nhs.uk/south/wp-content/uploads/sites/6/2017/07/national-stroke-strategy-2007.pdf. Accessed 06 April 2002.
4. National Institute for Health and Care Excellence (NICE). Stroke rehabilitation in adults: Clinical guideline [CG162]. 2013. https://www.nice.org.uk/guidance/CG162. Accessed 06 April 2020.
5. NHS England, National Stroke Service Model, National Stroke Programme, 2021 national-stroke-service-model-integrated-stroke-delivery-networks-may-2021.pdf (england.nhs.uk) Accessed 18 September 2021
6. NHS England. NHS Five Year Forward View: NHS England. 2014. https://www.england.nhs.uk/wp-content/uploads/2014/10/5yfv-web.pdf. Accessed 06 April 2020.
7. Intercollegiate Stroke Working Party. National clinical guideline for stroke. 4th ed. Royal College of Physicians. 2012. https://www.strokeaudit.org/Guideline/Historical-Guideline/National-Clinical-Guidelines-for-Stroke-fourth-edi.aspx. Accessed 27 May 2020.
8. Department for Work & Pensions and Department of Health (2017) Improving lives. The future of work, health and disability. London: Stationery Office. Available at: Improving Lives The Future of Work, Health and Disability (publishing.service.gov.uk) Accessed 18 September 2021.
9. Black CM, Frost D. Health at work - an independent review of sickness absence. Department for Work and Pensions. 2011. https://assets.publishing.service.gov.uk/government/uploads/system/uploads/attachment_data/file/181060/health-at-work.pdf. Accessed 06 April 2020.
10. Sinclair E, Radford K, Grant M, et al. Developing stroke-specific vocational rehabilitation: a soft systems analysis of current service provision. Disability and Rehabilitation; 2014; 36:409-17.
11. Royal College of Physicians, Sentinel Stroke National Audit Programme (SSNAP). Post-acute audit. In: Intercollegiate Stroke Working Party (ed.) 2015 Accessible at Sentinel Stroke National Audit Programme (SSNAP) | RCP London, Accessed 18 September 2021

12. Lock S, Jordan L, Bryan K, Maxim J. Work after stroke: focusing on barriers and enablers. Disability & Society. 2005; 20: 33–47
13. Royal College of Occupational Therapists (RCOT). Getting my life back: occupational therapy promoting mental health and wellbeing in England. RCOT. 2018. https://www.kmpt.nhs.uk/media/1059/getting-my-life-back_england.pdf. Accessed 12 May 2020.
14. Public Health England (PHE). Health matters: health and work. PHE. 2019. https://www.gov.uk/government/publications/health-matters-health-and-work/health-matters-health-and-work. Accessed 12 May 2020.Westerlind etc
15. NHS Outcomes Framework (NHS OF) - NHS Digital Summary Tables: NHS Digital. 2021. https://files.digital.nhs.uk/satatistical/nhs-outcomes-framwork. Accessed 18 September 2021.
16. Department for Work and Pensions (DWP) (2020). The employment of disabled people 2019. Available at: https://www.gov.uk/government/statistics/the-employment-of-disabled-people-2019, DWP 2020Accessed 18 September 2021
17. The Equality Act, (2010) accessible at; https://www.legislation.gov.uk/ukpga/2010/15/contents
18. Grant M. Developing, delivering and evaluating stroke specific vocational rehabilitation: a feasibility randomised controlled trial [PhD thesis on the Internet]. Nottingham, UK: University of Nottingham Repository. 2016. http://eprints.nottingham.ac.uk/35108/. Accessed 27 May 2020.

19. World Health Organisation, The International Classification of Functioning Disability and Health. 2001 Available at: International Classification of Functioning, Disability and Health (ICF) (who.int) (accessed 18 September 2021
20. Loisel P, Durand M-J, Berthelette D, et al. Disability prevention: New paradigm for the management of occupational back pain. Disease Management and Health Outcomes, 2001, 9(7): 351–360.
21. Fadyl J, McPherson KM, Schulter P, Turner-Stokes L. Development of a new tool to evaluate work support needs and guide vocational rehabilitation: The Work-ability Support Scale (WSS), Disability and Rehabilitation. 2014. 37:3, 247-258, DOI: 10.3109/09638288.2014.914586
22. Powers K, Clarke S, Cripps R, Holmes JA, Phillips J, Craven K, Farrin A, das Nair R, Radford KA, Developing and testing an implementation fidelity checklist for a complex vocational rehabilitation intervention, in preparation

For peer review only

Appendix II RETAKE OT Competency Marking Rubric

Criteria	Needs support	Competent	Highly competent
	≤49%<br><br>Demonstrates some understanding of ESSVR and its application in RETAKE. However, major deficits noted in VR knowledge, clinical reasoning and application. Requires additional individualised mentoring until next assessment.	50-69%<br><br>Understands ESSVR with some evidence of misinterpretation in its application in RETAKE. Ad hoc monitoring via group mentoring until next assessment.	≥70%<br><br>Fully understands ESSVR and its application in RETAKE.
**Knowledge of intervention processes, timeframes & documentation**<br><br>**(40% of total marks)**	Most answers were missing the required ESSVR components.	Some answers were missing the required ESSVR components.	Few, if any of the required ESSVR components were missing in the answers.
**Clinical reasoning – identification and analysis of salient work-related issues in the case study, to inform the design of an appropriate intervention (ESSVR) plan in the letter/report.**<br><br>**(50% of total marks)**	Limited identification of and/or limited analysis of work-related issues from the case study. None or few solutions for the work-related issues identified within the intervention plan(s). Significant gaps remain in problem-solving.	Some identification of and/or some analysis of work-related issues from the case study. A number of solutions for the work-related issues identified within the intervention plan(s) but a few gaps remain in problem-solving.	Identification and or analysis of all work-related issues from the case study. Comprehensive solutions for the work-related issues within the intervention plan(s).
**Written communication of work issues. Appropriate use of lay language in letter/report to ensure if it is fit for purpose & likely to gain reader engagement.**<br><br>**(10% of total marks)**	Letter/report lacks logical structure. Limited focus of work issue(s) addressed. Overuse of medical terminology. Little use of lay language to communicate issues. Information conveyed in a manner less likely to engage recipient.	Case study letter/report reasonably well structured. Mostly focussed on the work issue(s) being addressed. Minimal use of medical terminology. Good use of lay language to communicate issues. Information conveyed in a manner may to engage recipient.	Case study letter/report very well structured. Report fully focussed on work issue(s) addressed. Issues communicated clearly in lay language and without any use of medical terminology. Information conveyed in a manner likely to engage recipient.

RETAKE OT competency rubric Appendix II
